# Transmembrane Transport of Bicarbonate Unravelled**

**DOI:** 10.1002/chem.202100491

**Published:** 2021-05-01

**Authors:** Luis Martínez‐Crespo, Sarah H. Hewitt, Nicola Alessandro De Simone, Vladimír Šindelář, Anthony P. Davis, Stephen Butler, Hennie Valkenier

**Affiliations:** ^1^ Université Libre de Bruxelles (ULB) Engineering of Molecular NanoSystems, Ecole polytechnique de Bruxelles Avenue F.D. Roosevelt 50, CP165/64 1050 Brussels Belgium; ^2^ Loughborough University Department of Chemistry Epinal Way Loughborough LE11 3TU UK; ^3^ Masaryk University Department of Chemistry and RECETOX, Faculty of Science Kamenice 5 625 00 Brno Czech Republic; ^4^ University of Bristol School of Chemistry Cantock's Close Bristol BS8 1TS UK

**Keywords:** bicarbonate, fluorescent probes, ion transport, membranes, supramolecular chemistry

## Abstract

Anion receptors can be used to transport ions across lipid bilayers, which has potential for therapeutic applications. Synthetic bicarbonate transporters are of particular interest, as defects in transmembrane transport of bicarbonate are associated with various diseases. However, no convenient method exists to directly observe bicarbonate transport and study the mechanisms involved. Here, an assay is presented that allows the kinetics of bicarbonate transport into liposomes to be monitored directly and with great sensitivity. The assay utilises an encapsulated europium(III) complex, which exhibits a large increase in emission intensity upon binding bicarbonate. Mechanisms involving CO_2_ diffusion and the dissipation of a pH gradient are shown to be able to lead to an increase in bicarbonate concentration within liposomes, without transport of the anion occurring at all. By distinguishing these alternative mechanisms from actual bicarbonate transport, this assay will inform the future development of bicarbonate transporters.

## Graphical abstract

**A new assay to study bicarbonate transport** is reported, which is based on a europium complex that is encapsulated inside liposomes and reports changes in the intravesicular concentration of bicarbonate in a direct and highly sensitive way. This assay was used to reveal that synthetic anion transporters can act via different mechanisms: 1) actual transport of HCO_3_^−^ anions; 2) CO_2_ diffusion coupled to dissipation of the resulting transmembrane pH gradient.

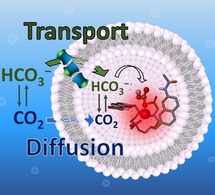

## Introduction

The transport of bicarbonate is crucial to many biological processes, such as the regulation of pH[^1^, ^2^] and the removal of metabolic waste.^[3]^ The development of synthetic HCO_3_^−^ transporters could contribute to the study and understanding of various diseases linked to mutations in HCO_3_^−^ transporting proteins, such as haemolytic anaemia, renal diseases, congenital chloride diarrhoea, and glaucoma.^[3]^ Furthermore, HCO_3_^−^ transporters have potential therapeutic applications and were reported to restore the properties of airway surface liquid in cystic fibrosis airway epithelial tissue.[^4^, ^5^]

Despite the importance of bicarbonate transport in health and disease, most research on synthetic anion transporters to date has focussed on chloride transport.[^6^, ^7^, ^8^, ^9^, ^10^] This is mainly driven by the ease by which Cl^−^ transport can be studied compared to that of HCO_3_^−^. Whereas Cl^−^ transport through the membranes of liposomes can be readily monitored by fluorescent probes or by ion selective electrodes (ISE),^[8]^ no equivalent methods for the study of HCO_3_^−^ transport exist. The pH sensitive probe HPTS has been widely used to study transport of many different anions and cations;^[11]^ however, this method cannot be readily adapted for the study of HCO_3_^−^ transport, due to the inherent pH variations in HCO_3_^−^ solutions over time.

**Figure 1:**
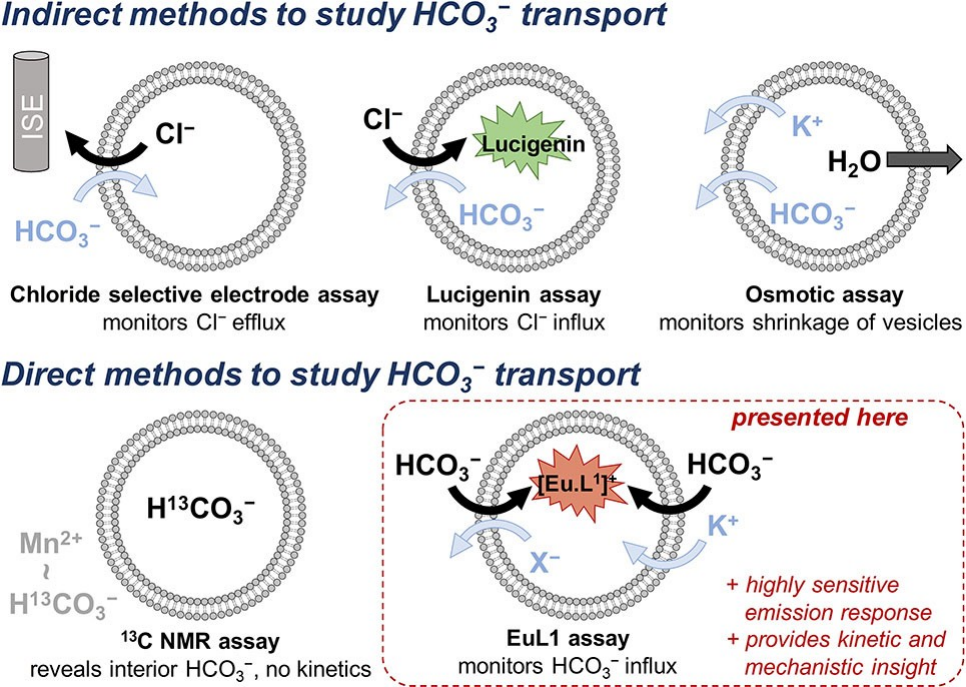
Existing and new methods to study HCO_3_^−^ transport.

Monitoring HCO_3_^−^ transport across lipid membranes remains a significant challenge. In the homeostasis of biological systems, the transmembrane transport of HCO_3_^−^ anions and the spontaneous diffusion of CO_2_ through membranes are two closely associated processes, which have clearly distinct roles.^[3]^ In model systems such as unilamellar vesicles (LUVs), it is not possible to distinguish between the actual transport of HCO_3_^−^ and mechanisms based on CO_2_ diffusion using current assays. Consequently, the exact mechanism(s) by which synthetic HCO_3_^−^ transporters operate remains ambiguous. Nonetheless, numerous reports on HCO_3_^−^ transporting anionophores exist,[^12^, ^13^, ^14^, ^15^, ^16^, ^17^, ^18^, ^19^, ^20^, ^21^] of which the first described a series of isophthalamides and the natural compound prodigiosin.^[12]^ However, indirect methods were employed to study the kinetics of HCO_3_^−^ transport by these and other compounds (Figure 1). In most cases, either the efflux of Cl^−^ out of liposomes was monitored with a chloride selective electrode,[^12^, ^13^, ^14^, ^15^, ^16^, ^17^] or the influx of Cl^−^ was followed with the fluorescent probe lucigenin[^18^, ^19^, ^20^] or SPQ.^[21]^ From the observed Cl^−^ transport it was concluded that an antiport process with HCO_3_^−^ must have taken place. Consequently, these methods are restricted to the study of Cl^−^/HCO_3_^−^ antiport only, and do not permit the study of exchange with any other anions, nor HCO_3_^−^ uniport. This limits the possibilities of studying and understanding HCO_3_^−^ transport.

The only existing method for the direct study of HCO_3_^−^ transport relies upon ^13^C NMR spectroscopy in combination with NaH^13^CO_3_ and a paramagnetic species, to distinguish interior from exterior isotopically labelled bicarbonate.[^5^, ^12^, ^13^, ^14^, ^21^] The major disadvantage of this method is the difficulty in monitoring transport processes over time (requiring 3–5 minutes per NMR spectrum), which precludes the accurate measurement of transport kinetics.^[22]^ More recently, an osmotic assay was reported where the efflux of HCO_3_^−^ by an anionophore is accompanied by the efflux of a cation (by a cationophore), resulting in an osmotic efflux of water, which can be observed as a change in the scattering intensity of the liposome dispersion.^[23]^ This is a promising strategy for studying HCO_3_^−^ uniport; however, the assay suffers from a low sensitivity and requires relatively large concentrations of transporter to be present in the membranes (∼10 mol%).

The limitations associated with current methods clearly call for a new assay that can report on HCO_3_^−^ transport directly, accurately and with high sensitivity. Fluorescence‐based transport assays offer a great sensitivity and can be readily implemented.[^24^, ^25^, ^26^] A fluorescence‐based assay in which the influx of HCO_3_^−^ can be monitored directly would thus surmount the current limitations, enabling an accurate comparison and quantification of rates of HCO_3_^−^ transport, and verification of the results obtained by indirect methods. Crucially, it would enable the mechanisms of transport to be elucidated unequivocally, including *i*) exchange processes of HCO_3_^−^ with different anions (antiport), *ii*) uniport of HCO_3_^−^, and *iii*) identification of actual transport of HCO_3_^−^ versus mechanisms based on CO_2_ diffusion. Such an assay requires a water‐soluble probe whose emission intensity changes in response to HCO_3_^−^ levels, whilst being unaffected by the presence of other anions and cations in the assay.

**Figure 2:**
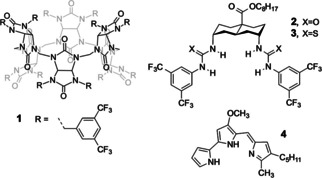
Structures of anionophores **1**–**4**.

The cationic europium complex [Eu.L^1^]^+^ previously developed by Butler for the purpose of detecting fluoride ions,^[27]^ satisfies these requirements. [Eu.L^1^]^+^ binds reversibly to HCO_3_^−^ in aqueous solution and shows an increase in Eu(III) emission intensity upon binding, particularly within the emission band centred at 615 nm. In contrast, [Eu.L^1^]^+^ has negligible responses to Cl^−^ and NO_3_^−^ and this made it an ideal candidate for the development of the transport assay. We present here the use of this emissive probe encapsulated in liposomes, to directly monitor the transport of HCO_3_^−^ across the lipid bilayers by fluorescence spectroscopy. We have used this new assay to study HCO_3_^−^ transport by a series of highly potent synthetic anionophores (**1**–**3**, Figure 2) and natural product prodigiosin (**4**), for which transport was previous observed indirectly using the lucigenin assay (Figure S1).[^18^, ^20^, ^28^]

This novel HCO_3_^−^ assay allows the study of the kinetics and mechanisms of HCO_3_^−^ transport by ionophores in unprecedented detail, as well as the comparison of various antiport and uniport processes. We have established that transporters **1**–**4** operate in different ways, and that only **1** is a “pure” HCO_3_^−^ carrier, transporting the anion without interference from other processes. Our results raise the distinct possibility that reported HCO_3_^−^ transporters might not transport the HCO_3_^−^ anion, but rather dissipate the pH gradient induced by CO_2_ diffusion. The assay represents a significant step forwards for identifying pure HCO_3_^−^ transporters and providing the mechanistic insight required to develop their potential biological applications.

## Results and Discussion

### Assay to monitor transport of bicarbonate directly

**Figure 3:**
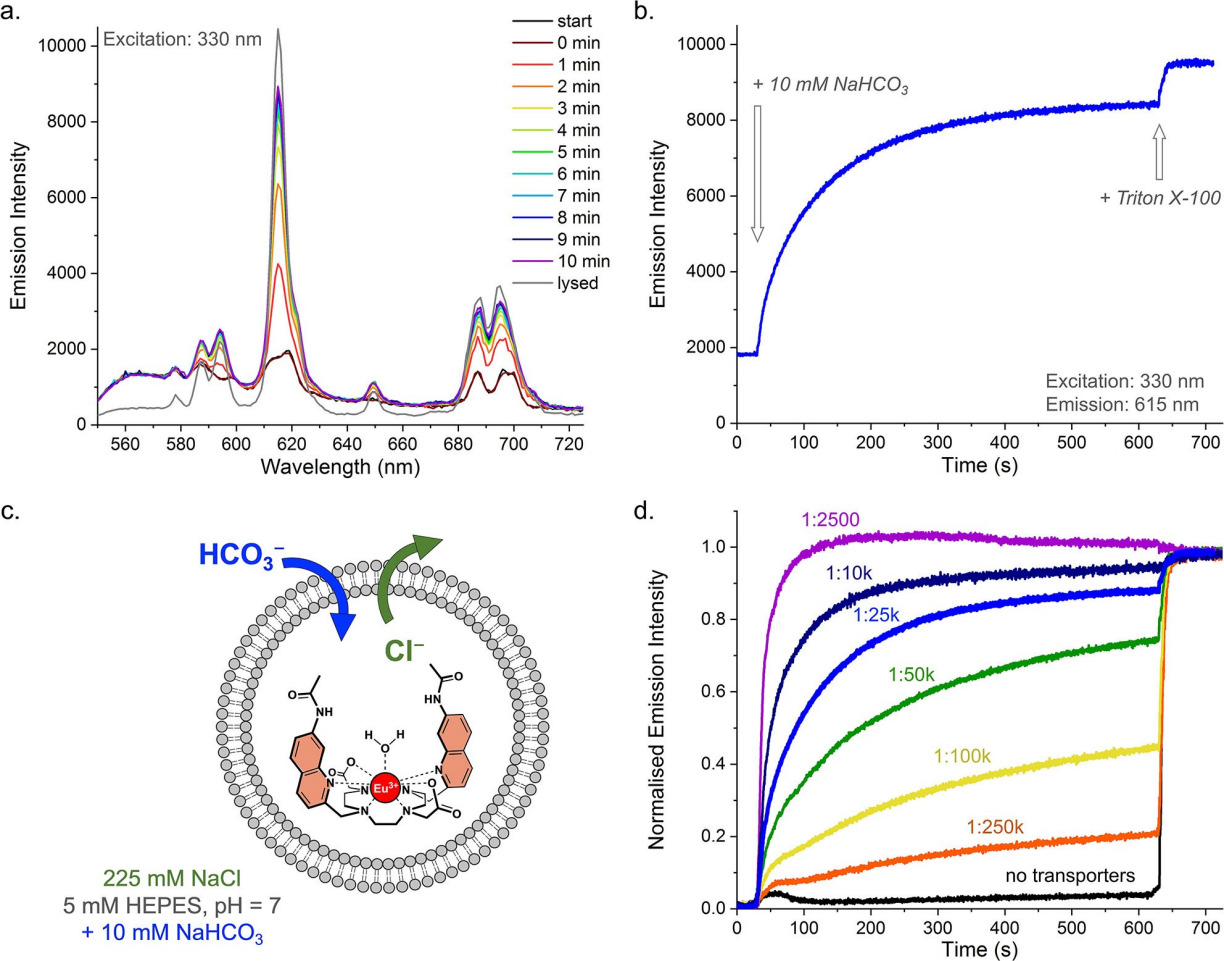
Transport of HCO_3_^−^ by anionophore **1** preincorporated in LUVs with the probe [Eu.L^1^]^+^ encapsulated (50 μM), suspended in 225 mM NaCl with 5 mM HEPES at pH 7.0 (interior and exterior), upon addition of 10 mM NaHCO_3_ after 30 seconds and lysis of the LUVs after 10 minutes: a. Emission spectra of [Eu.L^1^]^+^ recorded during the transport by **1** (at 1 : 25 k transporter to lipid ratio); b. Emission intensity at 615 nm monitored over time for the transport as in a.; c. Schematic representation of EuL1 assay to study transport of HCO_3_^−^; d. Normalised transport curves for anionophore **1** preincorporated at various anionophore to lipid ratios.

The cationic Eu(III) complex [Eu.L^1^]^+^ (Figure 3c) is based on a cyclen scaffold possessing two pendant quinoline arms that absorb UV light around 330 nm and transfer energy efficiently to the central Eu(III) ion, which emits red light in the range 570–720 nm.^[27]^ The Eu(III) probe has an open coordination site, occupied by a single water molecule in aqueous solution which quenches the Eu(III) emission significantly. In the presence of HCO_3_^−^, the coordinated water molecule is displaced upon binding of the hard oxyanion, resulting in a large enhancement in emission intensity (especially around 615 nm) and changes in spectral form (Figure 3a).^[29]^ The probe responds to physiologically relevant (millimolar) concentrations of HCO_3_^−^ and exhibits high selectivity over poorly coordinating anions that are commonly used in anion transport assays, including Cl^−^ and NO_3_^−^.^[27]^ The complex is also sensitive to hydroxide ions, and thus to pH, but this can be controlled readily with the use of a buffer (see ESI).

In order to use [Eu.L^1^]^+^ to monitor the transport of HCO_3_^−^, we encapsulated this probe into large unilamellar vesicles (LUVs) consisting of the lipids POPC and cholesterol in a 7 : 3 ratio and extruded these liposomes through a membrane with 200 nm pores, to obtain LUVs with an average hydrodynamic diameter of 183 nm (Figure S2). Liposomes of this diameter are routinely used for transport experiments by fluorescence spectroscopy and can be prepared reliably with a high degree of unilamellarity, in contrast to much larger vesicles, as used in the ^13^C NMR assay.^[12]^ An aqueous solution of 225 mM NaCl was present both interior and exterior to facilitate HCO_3_^−^/Cl^−^ exchange (antiport), which also contained 5 mM HEPES buffer to adjust the pH to 7.0 (Figure 3c). Anionophore **1** was preincorporated in the membrane of the LUVs and a NaHCO_3_ solution was added to create a HCO_3_^−^ concentration gradient of 10 mM (Figure S3). An increase in the intensity of the different emission bands of [Eu.L^1^]^+^ was observed (Figure 3a) upon the addition of NaHCO_3_. We chose to monitor the Δ*J*=2 emission band around 615 nm (see Figure 3b), as this showed the largest increase, in agreement with observations in titrations of [Eu.L^1^]^+^ with HCO_3_^−^.^[27]^ From here on, we will refer to these experimental conditions as the EuL1 assay.

The increase in the emission intensity over time following the addition of NaHCO_3_ indicates that HCO_3_^−^ has entered the liposomes. Since hardly any change in the emission intensity was observed in the absence of anionophore **1** (Figure 3d, black curve), we can conclude that bambusuril **1** transports HCO_3_^−^ into the liposomes and that the new EuL1 assay allows this process to be monitored. The concentration of **1** was varied (Figure 3d) and a clear increase in the rate of transport was observed for increasing concentrations of anionophore **1**. This shows that the EuL1 assay is highly sensitive and can be used to study the kinetics of HCO_3_^−^ transport. Furthermore, these results reinforce our previous findings that bambusuril **1** is a very potent HCO_3_^−^/Cl^−^ transporter,^[20]^ showing activity even at 1 : 250,000 ratio, which corresponds to 1.6 nM concentration and an average of two bambusurils per LUV.

### Differentiating the mechanisms of bicarbonate transport

**Figure 4:**
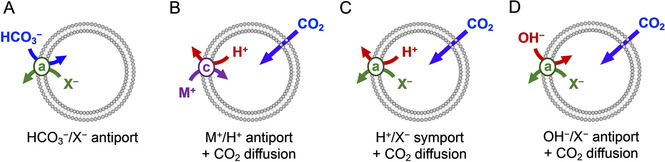
Different mechanisms by which apparent transport of HCO_3_^−^ could occur. In mechanism A, anionophore (a) exchanges HCO_3_^−^ for another anion – we refer to this as actual HCO_3_^−^ transport. Mechanisms B−D rely on the diffusion of CO_2_ coupled to transport of H^+^ or OH^−^ by cationophores (c) or anionophores to result in the net increase in HCO_3_^−^ concentration, without this anion crossing the membrane.

**Scheme 1:**
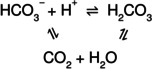
Representation of the interconversion between bicarbonate, carbonic acid, and carbon dioxide.

The processes by which actual and apparent HCO_3_^−^ transport can occur are schematically represented in Figure 4. The simplest mechanism for HCO_3_^−^ transport is the antiport process with another anion, such as Cl^−^ (Figure 4, mechanism A). However, we should consider that addition of a pulse of NaHCO_3_ to the exterior of the liposomes at pH<8 does not only create a gradient of HCO_3_^−^, but also of its conjugate acid H_2_CO_3_ ^[23]^ and of CO_2_, formed upon dehydration (Scheme 1).^[30]^ At equilibrium the concentration of CO_2_ is almost 1000‐fold higher than that of H_2_CO_3_ in aqueous salt solutions.^[31]^ Furthermore, it is well known that CO_2_ can diffuse spontaneously across the membranes of cells that play important roles in HCO_3_^−^ homeostasis,^[32]^ such as red blood cells and renal epithelial cells.^[3]^ Upon the addition of the HCO_3_^−^ pulse, CO_2_ could thus diffuse across the membranes of our liposomes. This increase in the concentration of CO_2_ inside the liposomes would result in an acidification of the interior, causing a pH gradient to build up, which would stop the diffusion of CO_2_. However, when transporters that can dissipate pH gradients are present in the membrane, the diffusion of CO_2_ can continue, leading to a net increase in HCO_3_^−^ concentration inside the liposomes, without this anion crossing the membrane (Figure 4B−D).

**Figure 5:**
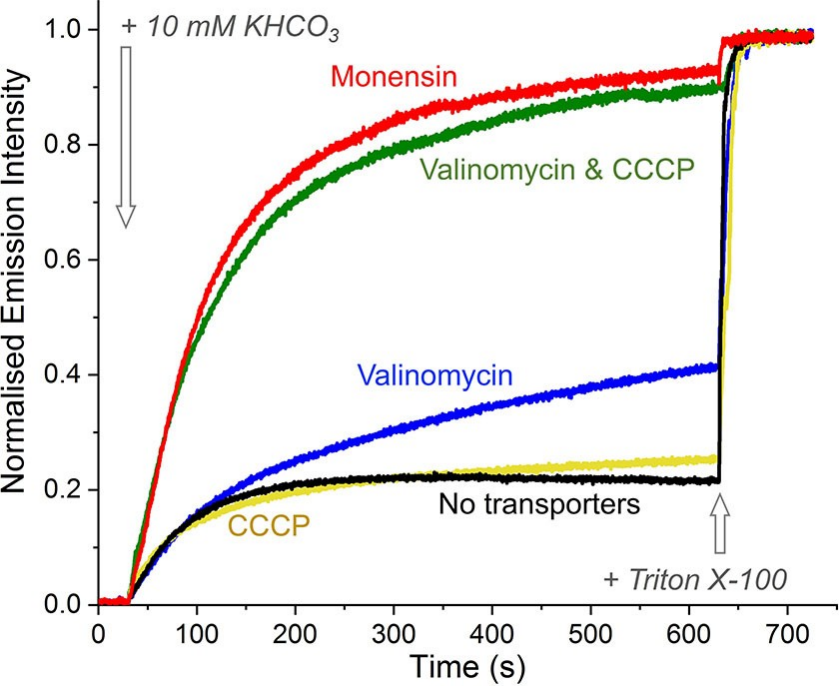
Increase in interior HCO_3_^−^ concentration as monitored by the EuL1 assay in 112 mM K_2_SO_4_ with 5 mM HEPES at pH 7, upon addition of 10 mM KHCO_3_ after 30 seconds. Different cation transporters were added to the LUVs at a transporter to lipid ratio of 1 : 1000.

We indeed found that the addition of the cationophore monensin (H^+^/M^+^ antiporter)[^33^, ^34^] to liposomes with [Eu.L^1^]^+^ encapsulated gave a clear response upon addition of a HCO_3_^−^ pulse (Figure 5, red curve). A similar response was observed when the combination of K^+^ transporter valinomycin^[35]^ and the protonophore carbonyl cyanide 3‐chlorophenylhydrazone (CCCP)^[36]^ were added (Figure 5, green curve), while those transporters added individually gave no significant response. Monensin gives similar transport curves in MCl, MNO_3_, and M_2_SO_4_ solutions (where M^+^ is Na^+^ or K^+^, see Figure S8 and 6a), in agreement with the anion independent CO_2_ diffusion mechanism B (Figure 4).

No systematic differences were observed between the experiments using either sodium or potassium salts. This could mean either that monensin performs H^+^/Na^+^ and H^+^/K^+^ antiport at identical rates, or that the formation^[30]^ and diffusion of CO_2_ are rate limiting in mechanism B. To distinguish between these possibilities, we varied the concentration of monensin. While decreasing the monensin to lipid ratio from 1 : 1000 to 1 : 10,000 gave a lower rate of transport, increasing to a ratio of 1 : 100 did not significantly impact the rate of transport (Figure S9). This confirms that the diffusion of CO_2_ is rate limiting in the observed increase in HCO_3_^−^ concentration within the liposomes and not the H^+^/M^+^ antiport by monensin.

**Figure 6:**
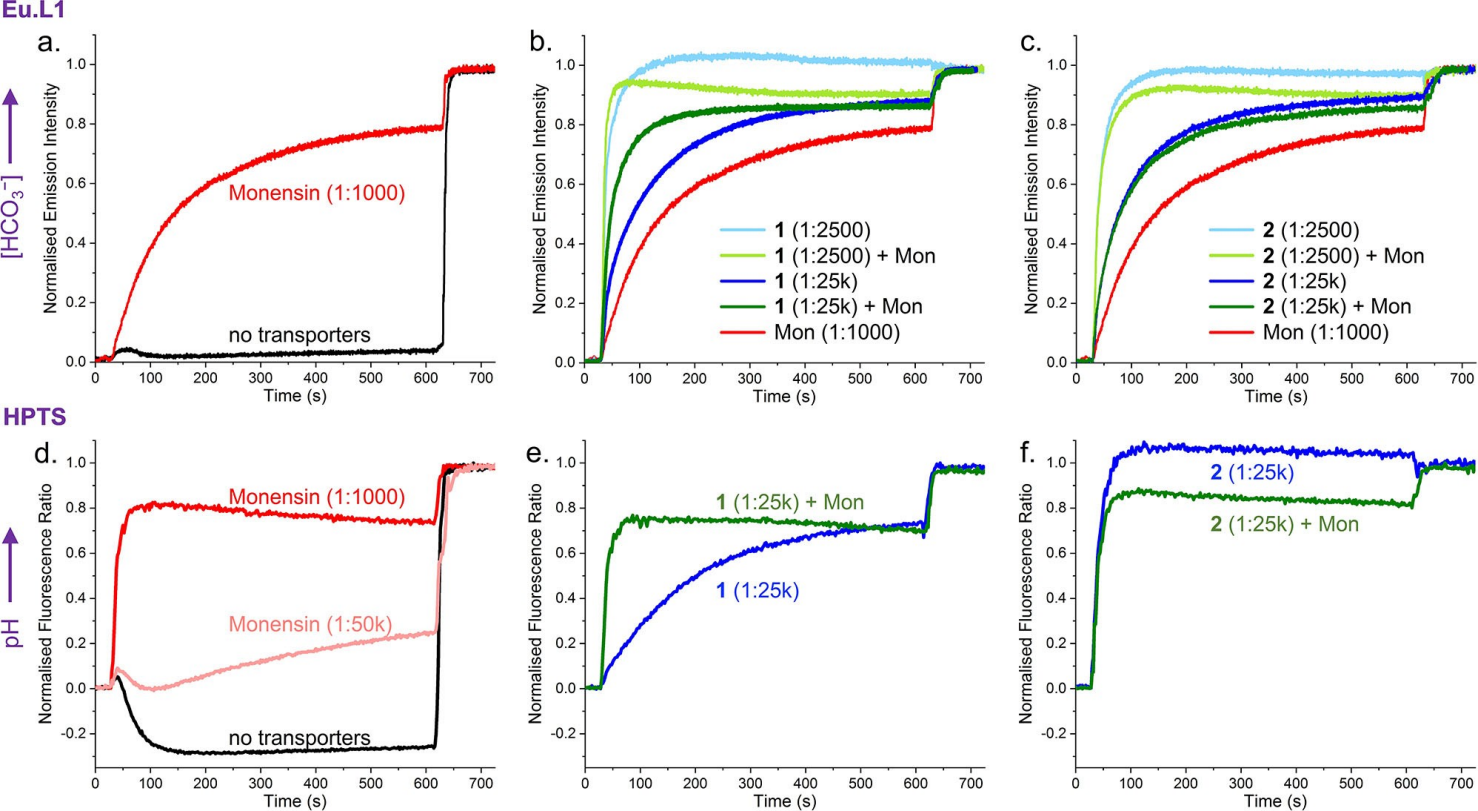
Increase in interior HCO_3_^−^ concentration as monitored using the EuL1 assay (a–c) or change of the interior pH as monitored using the probe HPTS (d–f) in 225 mM NaCl with 5 mM HEPES at pH 7, upon addition of 10 mM NaHCO_3_ after 30 seconds and lysis of the LUVs 10 minutes after that, to study transport by monensin (a,d), bambusuril **1** (b,e) and urea **2** (c,f). Monensin (1 : 1000 transporter to lipid ratio) was added to the experiments with anionophores **1** and **2**.

To verify if the pH inside the LUVs changes as expected upon diffusion of CO_2_ (in the absence of ionophores) or upon dissipation of the pH gradient by monensin, transport experiments were performed in which the pH sensitive probe HPTS was encapsulated instead of the bicarbonate sensitive probe [Eu.L^1^]^+^. All other conditions were identical to those used in the standard EuL1 assay (*i. e*., 225 mM NaCl, 5 mM HEPES, pH 7.0). The results in Figure 6d show that the addition of 10 mM NaHCO_3_ to LUVs with monensin (1 : 1000 ratio) results in a rapid increase of the pH to 7.4 (red curve), indicating the equilibration of the pH gradient caused by the addition of the basic solution of NaHCO_3_. In contrast, addition of NaHCO_3_ to LUVs without transporters results in an acidification of the interior (black curve), in agreement with the formation of carbonic acid upon diffusion of CO_2_. LUVs with a very low concentration of monensin (1 : 50,000) show an initial acidification of the interior due to CO_2_ diffusion, followed by a slow increase of the pH due to the H^+^/Na^+^ antiport by monensin. These experiments with HPTS confirm that the apparent transport of HCO_3_^−^ by monensin can be attributed to mechanism B. Furthermore, the pH equilibration by monensin at 1 : 1000 ratio (Figure 6d) is much faster than the apparent HCO_3_^−^ transport revealed by the EuL1 assay (Figure 6a), providing further evidence that CO_2_ diffusion (and/or formation^[30]^) is rate limiting in the apparent transport of HCO_3_^−^ by monensin (at 1 : 1000 ratio).

### Determining the transport mechanisms of different anionophores

Next, we studied the HCO_3_^−^ transport by anionophores **1**–**4** in the EuL1 assay in NaCl (blue curves in Figure 6b,c and S12). A clear increase of the emission intensity was observed for all anionophores, even at the relatively low concentration of 1 : 25,000 (transporter to lipid ratio). A key question we wished to address was whether the observed increase in HCO_3_^−^ in the liposomes is due to HCO_3_^−^/Cl^−^ antiport transport mechanism A, or rather by CO_2_ diffusion and pH gradient dissipation, as in mechanisms C and D (Figure 4).

Urea and thioureas with acidic N−H groups have been reported to not only transport anions, but also H^+^ (or OH^−^), and prodigiosin **4** is a known H^+^Cl^−^ transporter as well.^[37]^ Indeed, rapid pH equilibration was observed for anionophores **2**–**4** (blue curve in Figure 6f and S21). In contrast, bambusurils have an electron deficient cavity formed by twelve polarised methine C−H groups, which can neither be readily deprotonated nor interact strongly with OH^−^.^[38]^ Upon addition of NaHCO_3_ to liposomes with bambusuril **1**, a gradual increase in pH was observed, resembling the kinetics of the transport of the basic HCO_3_^−^ anion into the LUVs (blue curve in Figure 6e vs 6b). This result concurs with our previous finding that **1** is unable to dissipate pH gradients by HCl symport or Cl^−^/OH^−^ antiport.^[20]^ This excludes mechanisms C and D for this compound, leaving HCO_3_^−^/Cl^−^ antiport (A) as the only possible transport mechanism for **1**.

To distinguish the mechanisms involved in the apparent HCO_3_^−^ transport by the other compounds, we have made use of the limited rate of CO_2_ diffusion in the EuL1 assay, as observed from the experiments with monensin. For bambusuril **1**, addition of monensin gives a clear increase in the rate of transport as seen from the comparison of the green to the blue curves in Figure 6b and S14. This increase can be understood from the combined effect of mechanism A by **1** and mechanism B by monensin, leading to a higher rate of apparent transport of HCO_3_^−^ than by either of these two processes alone. In contrast, addition of monensin to LUVs with anionophores **2**–**4** did not increase the rate of transport, as shown for **2** in Figure 6c and for **3** and **4** in Figure S12. Because pH equilibration by these compounds is nearly instantaneous (Figure 6f and S21), the addition of a second pathway to dissipate the pH gradient (by monensin) will have no effect, as the overall rate of (apparent) HCO_3_^−^ transport will remain limited by CO_2_ diffusion. From this observation we can conclude that anionophores **2**–**4** primarily act via mechanism C or D.

To test if urea **2** and thiourea **3** can perform any HCO_3_^−^/Cl^−^ transport (mechanism A), we have increased the concentrations of **2** and **3** in the membranes of the liposomes to 1 : 2500 (transporter to lipid ratio). The light blue curves in Figure 6c and S12a show that this ten‐fold increase in transporter concentration leads to a significantly faster rate of (apparent) HCO_3_^−^ transport, and that this overall rate clearly exceeds rates of transport that are limited by CO_2_ diffusion (as observed in the curves for monensin ≥1 : 1000 ratio, see also Figure S13). From this we can conclude that HCO_3_^−^/Cl^−^ antiport mechanism A also takes place. These compounds dissipate the pH gradient faster than they transport HCO_3_^−^ and as a result C or D is the main mechanism, up to the point that CO_2_ diffusion becomes rate limiting, after which mechanism A contributes to the apparent HCO_3_^−^ transport. It is clear from these data that bambusuril **1** is the only “pure” HCO_3_^−^ transporter studied, which functions without interference from other processes.

### Bicarbonate uniport and antiport with nitrate

**Figure 7:**
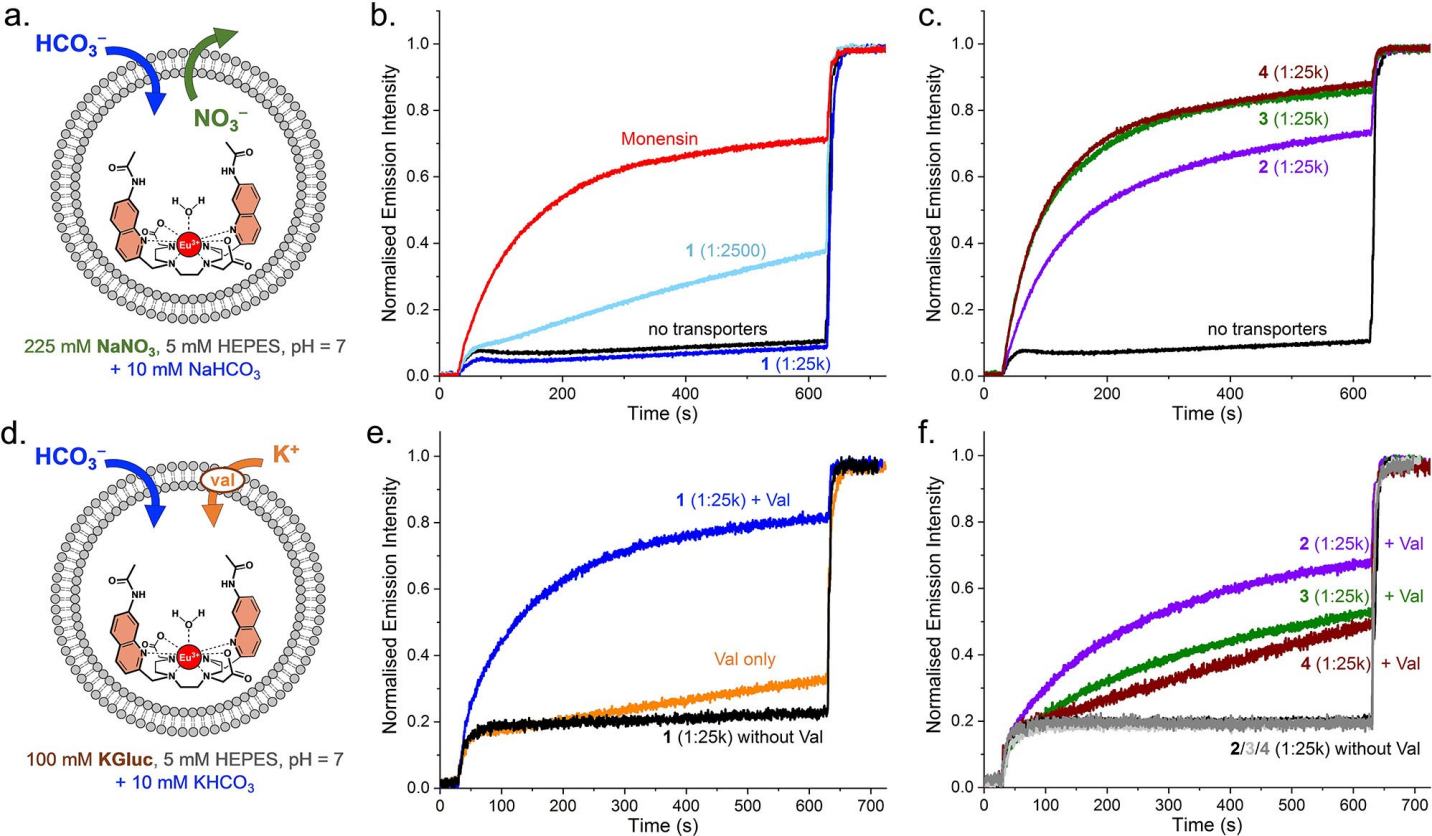
Increase in interior HCO_3_^−^ concentration in the presence of anionophores **1**–**4** monitored by the EuL1 assay in different salt solutions: a–c exchange with nitrate in 225 mM NaNO_3_ with 5 mM HEPES at pH 7, and d–f uniport in 100 mM KGluc with 5 mM HEPES at pH 7 in presence of valinomycin. 10 mM NaHCO_3_ (in b,c) or KHCO_3_ (in e,f) was added after 30 seconds and the LUVs are lysed after 10 minutes. The schematic representations in a and d only show the mechanisms based on actual HCO_3_^−^ transport, while mechanisms based on CO_2_ diffusion could also take place.

After demonstrating that our EuL1 assay can distinguish between actual and apparent HCO_3_^−^ transport mechanisms, we used the assay to study the exchange of HCO_3_^−^ with other anions, or uniport of HCO_3_^−^. Commonly employed indirect methods to study HCO_3_^−^ transport rely on the monitoring of Cl^−^ concentrations (Figure 1), preventing their use for studying exchange of HCO_3_^−^ and NO_3_^−^, or the uniport of HCO_3_^−^. In contrast, [Eu.L^1^]^+^ can operate in various salt solutions to study other processes than HCO_3_^−^/Cl^−^ exchange. Hence, we utilised the EuL1 assay in NaNO_3_ solution, to monitor HCO_3_^−^/NO_3_^−^ exchange (Figure 7a–c), and in a potassium gluconate (KGluc) solution in the presence of the K^+^ cationophore valinomycin, to study the uniport of HCO_3_^−^ (Figure 7d–f).

Compounds **2**–**4** were found to exhibit efficient (apparent) transport of HCO_3_^−^ in NaNO_3_ (Figure 7c) and the rates do not change upon addition of monensin (see Figure S16), similar to the results obtained for these compounds in NaCl, indicating that the same combination of mechanisms is occurring. However, bambusuril **1** showed no transport at a 1 : 25,000 ratio, and only very slow transport was observed when using a 10‐fold higher concentration of **1** (1 : 2500, Figure 7b). This slow HCO_3_^−^/NO_3_^−^ exchange by **1** resembles previous results reported for Cl^−^/NO_3_^−^ exchange, which was found to be 100‐fold slower than Cl^−^/HCO_3_^−^ exchange by this bambusuril.^[20]^ This large difference in Cl^−^/HCO_3_^−^ and Cl^−^/NO_3_^−^ exchange rates was explained by the very high affinity of **1** for NO_3_^−^ (K_a_=5×10^11^ M^−1^ in acetonitrile), which could prevent the release of this anion. In addition, it was proposed that simultaneous binding of a Cl^−^ and a HCO_3_^−^ anion in the bambusuril could facilitate the exchange of these anions.^[20]^ Even though the formation of an equivalent complex with NO_3_^−^ and HCO_3_^−^ simultaneously is possible, this does not appear to increase the rate of the exchange of these two anions by **1**. Instead, the very strong binding of NO_3_^−^ is the most probable cause for the low rates of HCO_3_^−^/NO_3_^−^ exchange by **1** (see also Figure S17).

This was further confirmed by the HCO_3_^−^ uniport experiment in KGluc (Figure 7e), where HCO_3_^−^ was efficiently transported by bambusuril **1**. In this experiment valinomycin transports K^+^ to compensate for the displacement of charge associated to the HCO_3_^−^ uniport, while the highly hydrophilic gluconate anion is not readily transported.^[23]^ Under these conditions, it is highly unlikely that an anion exchange process takes place and instead bambusuril **1** will have to release the strongly bound HCO_3_^−^ and return through the membrane without an anion bound.

In contrast, apparent transport of HCO_3_^−^ by thiourea **3** and prodigiosin **4** was much slower when tested in uniport conditions (Figure 7f), compared to in the presence of Cl^−^ or NO_3_^−^ (Figure 7c and S12). In NaCl and NaNO_3_ the apparent HCO_3_^−^ transport by these compounds was mainly attributed to H^+^/Cl^−^ or H^+^/NO_3_^−^ cotransport in combination with CO_2_ diffusion (mechanism C, or equivalent mechanism D). The poor rates of transport in KGluc indicate that these compounds are less efficient protonophores (H^+^ or OH^−^ transporters) than H^+^/Cl^−^ or H^+^/NO_3_^−^ cotransporters and that they are not efficient HCO_3_^−^ uniporters either. This result corroborates with other reports in which prodigiosin **4** was found to be a poor protonophore.^[37]^ The rate of apparent HCO_3_^−^ transport by urea **2** in KGluc is higher than those observed for **3** and **4** and the increase in the rate of transport with a higher concentration of **2** (Figure S18) indicates that **2** is able to perform actual HCO_3_^−^ uniport (see SI for further mechanistic discussions). Nonetheless, bambusuril **1** is clearly the most efficient HCO_3_^−^ uniporter tested.

### Quantification of transport rates

To verify the qualitative trends and comparisons described above, we fitted the transport data from the EuL1 assay with single and double exponential functions, to obtain half‐lives and initial rates respectively (see SI for details). Due to the slight differences observed in the equilibration levels of the different transport curves after normalisation and the effect of pH on the emission levels (see SI for a discussion), half‐lives are more reliable to compare transport data, as these values indicate the time required to reach half of the final transport level and are thus a measure of how fast equilibrium is reached, independent of absolute emission values. The obtained values for the half‐lives are given in Table 1 (see Table S1 for additional data). In NaCl, the comparison of half‐lives of transport by **1** with and without monensin clearly shows that equilibrium is reached much faster in the presence of monensin (Table 1 and Figure S15), confirming the additivity of mechanisms A and B as discussed above. In contrast, the half‐lives for **2**–**4** are nearly identical in the presence and absence of monensin, both in NaCl and in NaNO_3_.

**Table 1:** Performance of anionophores **1**–**4** in the EuL1 assay.

Salt	Aniono‐ phore	Concentration (anionophore: lipid)	Half‐life (s)^[a]^ without monensin	Half‐life (s)^[a]^ with monensin
NaCl	None		*	82
	**1**	1 : 2500	10	4
	**1**	1 : 25 k	64	21
	**2**	1 : 2500	12	11
	**2**	1 : 25 k	51	50
	**3**	1 : 2500	12	11
	**3**	1 : 25 k	46	47
	**4**	1 : 25 k	59	74
NaNO_3_	None		*	81
	**1**	1 : 2500	*	67
	**1**	1 : 25 k	*	85
	**2**	1 : 2500	45	40
	**2**	1 : 25 k	89	85
	**3**	1 : 2500	16	14
	**3**	1 : 25 k	65	59
	**4**	1 : 25 k	61	68
KGluc^[b]^	None		*	124
	**1**	1 : 25 k	83	38
	**2**	1 : 25 k	140	45
	**3**	1 : 25 k	180	42
	**4**	1 : 25 k	*	n.d.

[a] Calculated from a single exponential fit of the transport curve, see ESI for details. [b] Transport in KGluc was studied in presence of valinomycin. * Transport was absent or too slow to quantify. n.d.=not determined.

Table 1 also shows that the overall half‐lives obtained from the apparent HCO_3_^−^ transport by anionophores **1**–**4** (in absence of monensin) are rather similar in NaCl. However, the different pH profiles could affect this comparison (see SI Section 2.10) and it would thus be better to compare the different transporters in the presence of monensin. Under those conditions, CO_2_ diffusion‐based mechanisms contribute to the transport for all the compounds, but as this process has a limited and thus constant rate, the differences in half‐lives between anionophores **1**–**4** (in presence of monensin) can be attributed to the differences in rates of HCO_3_^−^/Cl^−^ antiport (mechanism A) by the anionophores. In this comparison, bambusuril **1** is clearly the most active ionophore for HCO_3_^−^/Cl^−^ antiport. Bis‐urea **2** and bis‐thiourea **3** show similar rates of transport and are slightly more active than prodigiosin **4**, for which the half‐life is very close to that of transport by monensin alone.

In contrast, in NaNO_3_, transport by bambusuril **1** was too slow to be quantified, while addition of monensin resulted in half‐lives that were identical or slightly lower than for monensin alone (for the lower and higher concentration of **1**, respectively). Half‐life values also indicate that apparent HCO_3_^−^ transport in NaNO_3_ by **2** is a bit slower than in NaCl, and that this is not the case for **3** and **4**, which show similar rates in both salt solutions.

The values of half‐lives obtained in KGluc and in presence of valinomycin clearly demonstrate that **1** is a much better HCO_3_^−^ uniporter than any of the other transporters. Again, transport by **1** can be enhanced by adding both valinomycin and monensin due to an additivity of mechanisms. In contrast, while rates of **2** and **3** in presence of both valinomycin and monensin are faster than with valinomycin only (Table 1), these rates are the same as with monensin only (see Table S2), indicating that these compounds could perform HCO_3_^−^/H^+^ symport (see SI for further discussion).

### Comparison of the EuL1 assay with existing methods

Our EuL1 assay for monitoring HCO_3_^−^ transport directly overcomes numerous disadvantages of existing methods, while combining their advantages, to offer a complementary tool in anion transport research (Figure 1). The only other direct HCO_3_^−^ transport assay is based on ^13^C NMR spectroscopy,^[12]^ which suffers from low sensitivity and poor time resolution. Furthermore, we observed that the cationophore monensin also gives a positive response in the ^13^C NMR assay for HCO_3_^−^/Cl^−^ transport (Figure S22), which demonstrates that this assay cannot distinguish between CO_2_ diffusion‐based mechanisms and actual HCO_3_^−^ transport. Indirect assays, such as ISE and lucigenin assays,[^12^, ^13^, ^14^, ^15^, ^16^, ^17^, ^18^, ^19^, ^20^] have not been able to provide mechanistic insights, nor allow comparisons between various HCO_3_^−^ antiport and uniport processes. The osmotic HCO_3_^−^ uniport assay is the only method reported so far that showed diffusion of neutral HCO_3_^−^‐based species in combination with H^+^ transport by monensin,^[23]^ in agreement with our findings. However, the drawbacks of the osmotic assay are the very high concentrations of ionophores required due to the low sensitivity and the lower compatibility of the assay with preincorporated lipophilic transporters in the membrane during the preparation of the LUVs.

We have exploited the attractive features of the EuL1 assay to discover that bambusuril **1** can efficiently perform HCO_3_^−^/Cl^−^ antiport and HCO_3_^−^ uniport, while bisurea **2**, thiourea **3**, and prodigiosin **4** mainly combine CO_2_ diffusion and pH gradient dissipation, leading to *apparent* HCO_3_^−^ transport. Compounds **1**–**4** have previously been shown to act as HCO_3_^−^ transporters in the lucigenin assay[^18^, ^20^] and prodigiosin **4** also in the ISE and ^13^C NMR assays.^[12]^ However, those experiments could not distinguish between actual and apparent transport of HCO_3_^−^ anions. Notably, most of the HCO_3_^−^ transporters reported in the literature resemble compounds **2**–**4** and can transport H^+^ or OH^−^.^[37]^ This transport activity combined with CO_2_ diffusion could be the mechanism of apparent HCO_3_^−^ transport for many reported compounds. It is striking that selectivity for transport of Cl^−^ and NO_3_^−^ over HCO_3_^−^ has been reported for only two compounds, a biotinuril macrocycle and a bis‐triazole,[^39^, ^40^] which do not have acidic protons and are thus likely to be poor H^+^ and OH^−^ transporters.

Our results imply that only for compounds that show apparent HCO_3_^−^ transport without dissipating pH gradients (such as **1**) and for very potent anion transporters for which the rate of total apparent HCO_3_^−^ transport surpasses the limited rate of CO_2_ diffusion (**2** and **3**), we can conclude with certainty that these can act as actual anionophores for HCO_3_^−^. This should be taken into account in the future development of HCO_3_^−^ anionophores and can be readily verified with the EuL1 assay.

## Conclusion

We have developed a new assay to directly monitor the transport of HCO_3_^−^ into liposomes by fluorescence spectroscopy, using the encapsulated europium complex [Eu.L^1^]^+^ that provides a luminescence increase upon binding HCO_3_^−^. This assay provides a rapid and highly sensitive signal that enables anion transport kinetics to be determined and low concentrations of anionophores to be used. By combining anionophores with monensin in this direct and sensitive assay, it was possible to distinguish *actual transport* of HCO_3_^−^ anions from alternative mechanisms based on CO_2_ diffusion, which lead to an increase of HCO_3_^−^ concentration in the liposomes without this anion crossing the membrane.

Our assay provides unprecedented insight into the mechanisms of HCO_3_^−^ transport by anionophores and leads to the conclusion that we should doubt if many of the reported HCO_3_^−^ transporters are actually capable of transporting this anion, or that they rather operate by dissipating the pH gradient resulting from CO_2_ diffusion. Furthermore, the versatility of the assay compared to all existing assays was demonstrated by comparing HCO_3_^−^/Cl^−^ and HCO_3_^−^/NO_3_^−^ antiport and HCO_3_^−^ uniport processes for the first time.

We are convinced that the new opportunities provided by this assay to study transport of HCO_3_^−^ efficiently and in new mechanistic detail will contribute to the further development of HCO_3_^−^ transporters for biomedical purposes, such as channel replacement therapies.[^6^, ^41^] As the anion binding properties of lanthanide probes such as [Eu.L^1^]^+^ can be tuned for different anions,[^29^, ^42^] the assay developed in this work could also be readily adapted to study transport of other anions. Furthermore, our work will also inform the future design of Eu(III) probes capable of monitoring spatio‐temporal HCO_3_^−^ dynamics within living cells. Indeed, a structurally related Eu(III) complex has already been shown to enter living cells and localise to specific subcellular compartments.^[43]^ This feature, combined with the long luminescence lifetime of [Eu.L^1^]^+^ and its derivatives augurs well for cellular imaging of HCO_3_^−^ transport with high signal‐to‐noise, using time‐gated fluorescence microscopy.

## Conflict of interest

The authors declare no conflict of interest.

## Supporting information

As a service to our authors and readers, this journal provides supporting information supplied by the authors. Such materials are peer reviewed and may be re‐organized for online delivery, but are not copy‐edited or typeset. Technical support issues arising from supporting information (other than missing files) should be addressed to the authors.

Supplementary
