# Supplementary material for: Transmembrane Transport of Bicarbonate Unravelled
Source: Chemistry. 2021 May 1;27(26):7367–75. doi: 10.1002/chem.202100491 (PMC8251953; doi:10.1002/chem.202100491)
Supplement: Supplementary file 1 — Supplementary [file CHEM-27-7367-s001.pdf]

Chemistry–A European Journal

Supporting Information

VIP

Transmembrane Transport of Bicarbonate Unravelled**

Luis Martínez-Crespo, Sarah H. Hewitt, Nicola Alessandro De Simone, Vladimír Šindelář, Anthony P. Davis, Stephen Butler,* and Hennie Valkenier*

CONTENTS

1 CHLORIDE/BICARBONATE EXCHANGE IN THE LUCIGENIN ASSAY 2

2 TRANSPORT EXPERIMENTS WITH THE EU.L1-BASED ASSAY 3

2.1 Procedures for the transport experiments with the Eu.L1 assay 3
General procedure for the preparation of liposomes for transport measurements 3
General procedure for bicarbonate transport measurements with Eu.L1 3
Fitting of the data 4

2.2 DLS analysis of the large unilamellar vesicles 5

2.3 Optimisation of the EuL1 assay 6

2.4 Control experiments in the EuL1 assay 7
Increase of bicarbonate concentration and not only of pH 7
Reproducibility and stability over time 7
Compatibility of the assay with different lipids 8

2.5 Transport by monensin and other cation transporters 9
Transport by different cationophores and gramicidin in K_2SO_4 9
Transport by monensin in different salt solutions 10
Transport with different concentrations of monensin 11
Transport at different concentrations of buffer and at different pH 11

2.6 Bicarbonate transport by anionophores in NaCl solutions 13
Bicarbonate transport by anionophores 3 and 4 in NaCl, in absence and presence of monensin 13
Bicarbonate transport by anionophores 1 and 2, with different concentrations of monensin 13
HCO_3^-/Cl^- exchange by bambusuril 1, in absence and presence of monensin 14

2.7 Bicarbonate transport by anionophores in $NaNO_3$ solutions 15

2.8 Bicarbonate uniport by anionophores in KGluconate solutions 17

2.9 Quantification of rates of transport 19

2.10 Titrations of Eu.L1 with $NaHCO_3$ 21
Titration under standard transport conditions (Sample 1) 21
Titration at pH higher than the standard (Sample 2) 21
Titration at constant pH (Sample 3) 21
Data treatment 21

3 TRANSPORT EXPERIMENTS WITH HPTS 23

3.1 Procedures for the transport experiments with HPTS 23

3.2 Additional results for the transport experiments with HPTS 23

4 TRANSPORT EXPERIMENTS WITH ^{13}C NMR SPECTROSCOPY 24

5 REFERENCES 26

1 Chloride/Bicarbonate exchange in the lucigenin assay

The transporters that were chosen for the studies in the new EuL1 assay had previously shown to be active Cl^-/HCO_3^- exchangers in the lucigenin assay. In this assay the influx of Cl^- is monitored, making it an indirect method to study the transport of HCO_3^-.[1,2] The performance of transporters **1-4** in this lucigenin assay is shown in Figure S1 for comparison.

The synthesis of compounds **1**,[1] **2**,[2] and **3**[3] has been reported and experimental details for the lucigenin assay can be found in reference 1.

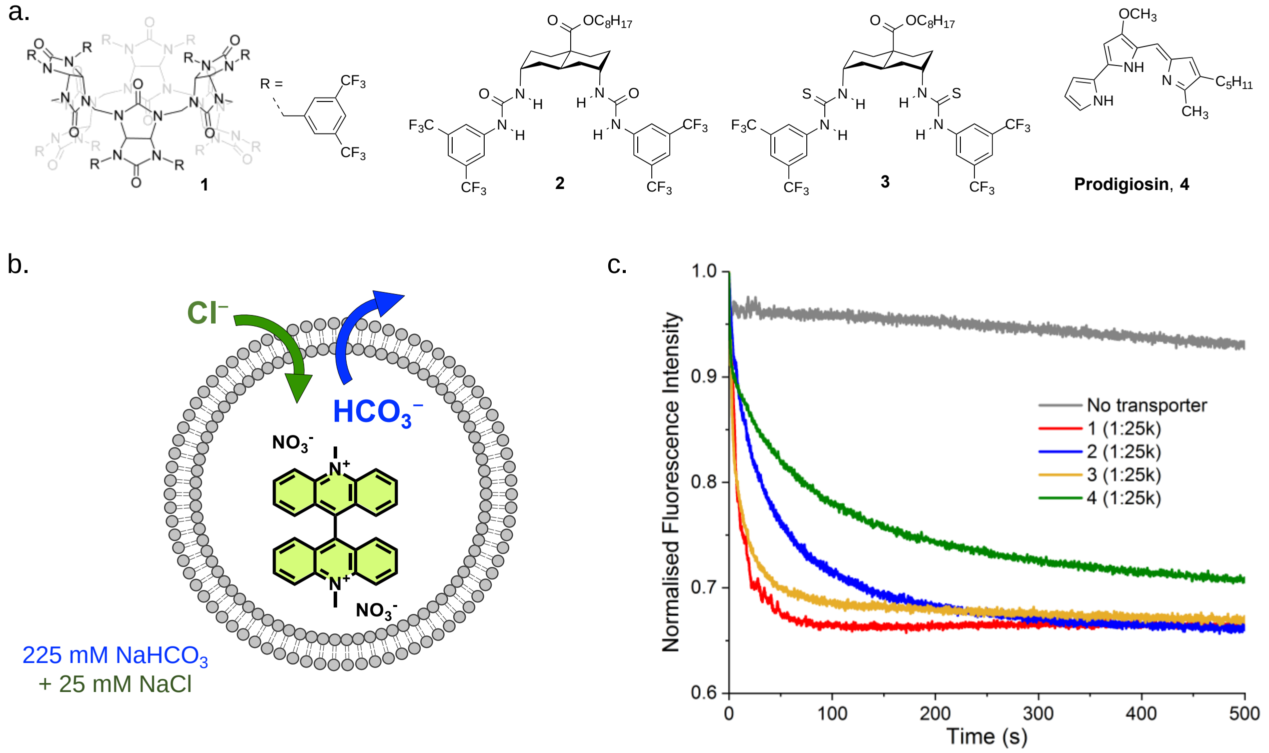

Figure S1a. Anion transport by transporters **1**-**4**, b. Schematic representation of the lucigenin assay, c. Chloride transport by transporters **1**-**4** (transporter:lipid ratio of 1:25k) into LUVs of POPC:cholesterol (70:30) suspended in 225 mM $NaHCO_3$ solution (interior and exterior, pH 8), monitored by the quenching of the fluorescence of encapsulated lucigenin upon addition of 25 mM NaCl.

2 Transport experiments with the Eu.L1-based assay

2.1 Procedures for the transport experiments with the Eu.L1 assay

General procedure for the preparation of liposomes for transport measurements

Lipid solutions of 1-Palmitoyl-2-oleoyl-*sn*-glycero-3-phosphocholine (POPC) and cholesterol were prepared using chloroform that had been deacidified by passage through a column containing basic alumina. All aqueous solutions were prepared using deionised water that had been passed through a Millipore filtration system. Liposomes were prepared in aqueous salt solutions of 225 mM (cation concentration) and buffered to pH 7 with 5 mM HEPES (unless indicated otherwise).
The synthesis and characterisation of the probe $[Eu.L^1]^+$ have been reported previously.[4]

POPC and cholesterol solutions (10-20 mM) in deacidified chloroform were combined with a solution of anionophore in organic solvent (0.1 mM **1** in acetonitrile, **2** and **3** as solutions in methanol) in a 5 mL round bottom flask. The volumes of the aliquots were calculated from the concentrations of the lipid solutions to obtain a POPC to cholesterol ratio of 7:3 (for instance by combining 7 µmol POPC and 3 µmol cholesterol) and the desired anionophore to lipid ratio, as specified in the experiments. The solvents were evaporated under a flow of nitrogen and the resulting lipid film was dried under high vacuum for at least 1 h.
The lipid film was then hydrated with 500 µL of an aqueous solution of the probe $[Eu.L^1]^+$ (50 µM, prepared from a 4.8 mM stock solution in methanol) in a solution of the desired buffer. The resulting mixture was sonicated for 30 s and stirred for 1 h to give heterogeneous vesicles. Multilamellar vesicles were disrupted by 10 freeze-thaw cycles. The mixture was diluted to 1 mL (by adding 0.5 mL of buffer solution) and extruded 29 times through a polycarbonate membrane with 200 nm pores in a mini-extruder (Avestin LiposoFast-Basic). The external probe was removed by passing the liposomes through a pre-packed size exclusion column (containing 8.3 mL Sephadex G-25 medium), eluted with buffer solution.[i] The collected large unilamellar vesicles were further diluted with buffer solution to obtain total lipid concentration of 0.4 mM and used for transport measurements the same day.

General procedure for bicarbonate transport measurements with Eu.L1

3.00 mL of this liposome solution was placed in a quartz cuvette with a small stir bar and the temperature was allowed to stabilize at 25 ˚C for 3-5 min. inside the sample compartment of a Fluoromax-4 spectrometer, that was equipped with a 420 nm cut-off filter between the sample and the detector. Emission spectra were recorded from 550-725 nm (3 nm slits, 1 nm interval, 0.3 s integration time) with excitation at 330 nm (10 nm slits). During transport measurements the emission intensity at 615 nm (3 nm slits) was monitored over time (15 minutes, 0.2 s interval) with excitation at 330 nm (10 nm slits).
If cation transporters (monensin, valinomycin, gramicidin, or CCCP) or prodigiosin were used, then these were added to the liposomes as 5 µL of solution in methanol (0.24 mM to create a 1:1000 cation transport to lipid ratio, unless stated otherwise) 5 minutes before the addition of the bicarbonate.
For transport measurements 38 µL of $MHCO_3$ (0.8 M, in 225 mM salt solution) was added to the liposomes 30 s after the start of the emission recording, to create a HCO_3^- concentration gradient of 10 mM. The emission intensity was measured for another 10 minutes, followed by lysing of the

[i] The SEC columns could be reused, but required rinsing with large amounts of 0.1 M Na_2SO_4 solution (~300 mL) to remove any remaining traces of the probe

liposomes by addition of 50 µL of Triton X-100 (5% $^{w}/_{w}$ in water). Three runs were performed per experiment (unless indicated otherwise).
The averaged data were normalised from 0 (before the addition of $MHCO_3$) to 1 (after lysis).

Fitting of the data

The averaged and normalised curves were fitted in Origin 2019b after removing the first 30 seconds of data (so that the $MHCO_3$ is added at t = 0 s), after which data after 500 s were removed (leaving the data measured at t = 30-530 s).

The normalised transport curves were fitted to a single exponential function to obtain half-lives:

$$F = y - ae^{-bt}$$

The initial values used for the fit parameters were y = 0.8, a = 0.8, b = 0.01 and not boundaries were set.
The half-life $t_{½}$ was calculated from fit parameter b using:

$$t_{½} = \frac{\ln(2)}{b}$$

The normalised transport curves were fitted to a double exponential function to obtain initial rates of transport:

$$F = y - ae^{-bt} - ce^{-dt}$$

The initial values used for the fit parameters were y = 1, a = 0.8, b = 0.05, c = 0.2, d = 0.01. All parameters were set to all be ≥ 0 and y, a, and $c \leq 1$.

The initial rate I was calculated from fitted parameters a, b, c, and d using:

$$I = ab + cd$$

Note that in the case of very low rates of transport, I cannot be determined accurately, as the fitting will be dominated by the very small initial response, which results in an overestimation of I. In the extremely high rates of transport, the equilibrium level upon transport can be briefly surpassed, which complicates the fitting of the curve. Therefore, only the first 60 s of the transport process were fitted with a double exponential function if $I > 0.06$ s^{-1} (which was the case for 1:2500 anionophore to lipid ratios in NaCl).

2.2 DLS analysis of the large unilamellar vesicles

The size of the liposomes was verified by Dynamic Light Scattering (DLS) measurements on a Malvern Zetasizer Ultra at 25 ˚C, using disposable cuvettes. Standard parameters were used for the liposomes (Refractive index 1.45) and the parameters for the aqueous NaCl buffer used were obtained from reference 5 (viscosity of 0.91 mPa·s and refractive index 1.33).

The average intensity DLS data from 6 different batches of LUVs are plotted in Figure S2 and Z-average values fall in the range of 177 to 190 nm, with an average of 183 nm for the hydrodynamic diameter of the LUVs.

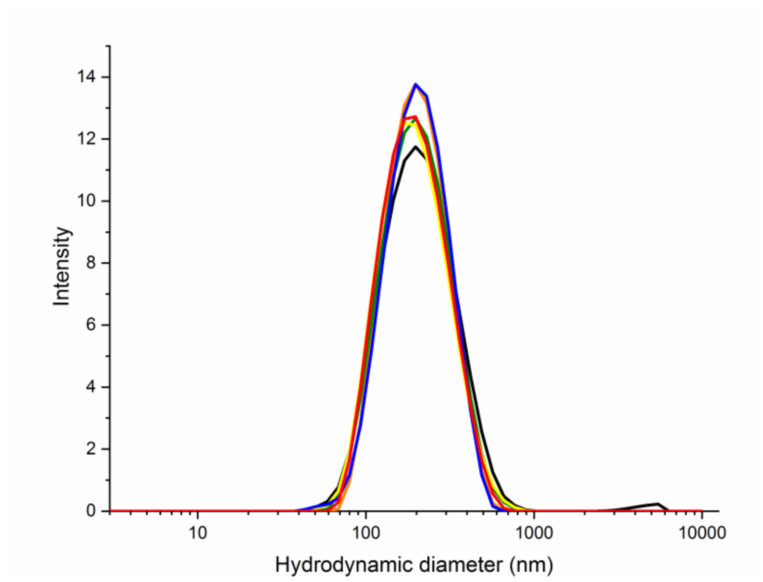

Figure S2. DLS data for 6 batches of LUVs with probe $[Eu.L^1]^+$ encapsulated and suspended in 225 mM NaCl with 5 mM HEPES at pH7 (each curve corresponds to the average of three consecutive measurements).

2.3 Optimisation of the EuL1 assay

As the emission response of the probe [**Eu.L^1**]$^+$ is based on a binding process of HCO_3^-, we have verified what concentration of HCO_3^- could be added to have a clear response, while avoiding approaching saturation of the probe with the anionic guest. For this we have prepared LUVs with 50 µM probe [**Eu.L^1**]$^+$ and compound **1** preincorporated at 1:2500 transporter to lipid ratio, as described in Section 2.1. Instead of adding $NaHCO_3$ as a single pulse, we have added 8 times 19 µL of 0.8 M $NaHCO_3$, corresponding to an increase of the $NaHCO_3$ concentration by 5 mM per addition. After each addition, we waited 3 minutes for transport to occur, which allowed to estimate the final emission levels reached upon addition of different concentrations of HCO_3^- in the transport assay (Figure S3).

While addition of 25 mM $NaHCO_3$ resulted in close to saturation of the probe, upon addition of 10 mM $NaHCO_3$ the probe gives a strong response, but is still far from saturation, ensuring a good sensitivity for the different concentrations of HCO_3^- up to this final concentration. For this reason, we have worked with a pulse of 10 mM $MHCO_3$ in all further experiments. See Section 2.6 for further binding studies with the probe [**Eu.L^1**]$^+$ and HCO_3^-.

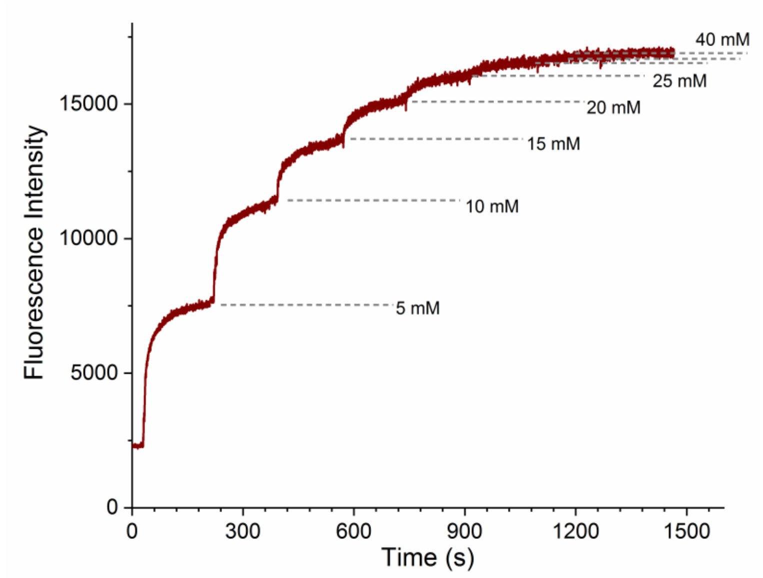

Figure S3. Transport of HCO_3^- by anionophore **1** (preincorporated at 1:2500 transporter to lipid ratio) into LUVs as monitored by the EuL1 assay in 225 mM NaCl with 5 mM HEPES at pH 7, upon 8 additions of 5 mM $NaHCO_3$.

Further optimisation of the assay involved the choice of buffer concentration and pH, which is discussed in Section 2.5.

2.4 Control experiments in the EuL1 assay

Increase of bicarbonate concentration and not only of pH

To demonstrate that, although the probe $[Eu.L^1]^+$ can show a small increase of emission intensity upon increasing pH in absence of HCO_3^-,[4] the response observed in our Eu.L1 assay is not due to the increase of the pH, an experiment was performed in which 2 mM NaOH was added instead of 10 mM $NaHCO_3$ to LUVs with monensin. While the increase in pH upon the addition of NaOH was slightly higher (7.0 to 7.7) than upon addition of 10 mM $NaHCO_3$ (7.0 to 7.5), the increase in emission intensity was much smaller (Figure S4).

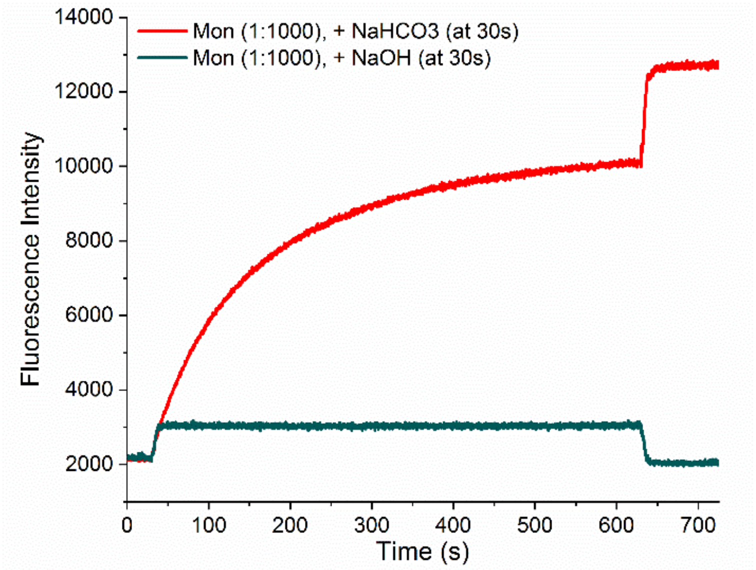

Figure S4. Transport as monitored by the Eu.L1 assay in 225 mM NaCl with 5 mM HEPES at pH 7, upon addition of 10 mM $NaHCO_3$ (resulting in an exterior pH of 7.5; red curve) or 2 mM NaOH (resulting in an exterior pH of 7.7; dark cyan) after 30 seconds. Monensin was added to the LUVs 5 minutes before addition of the basic solution at a transporter to lipid ratio of 1:1000. The LUVs were lysed 10 minutes after the addition of $NaHCO_3$ or NaOH.

Reproducibility and stability over time

The EuL1 assays shows no significant variation in fluorescence levels, neither before nor after addition of $NaHCO_3$, over the course of several hours, indicating that the probe $[Eu.L^1]^+$ is stable inside the liposomes and does not leach out.

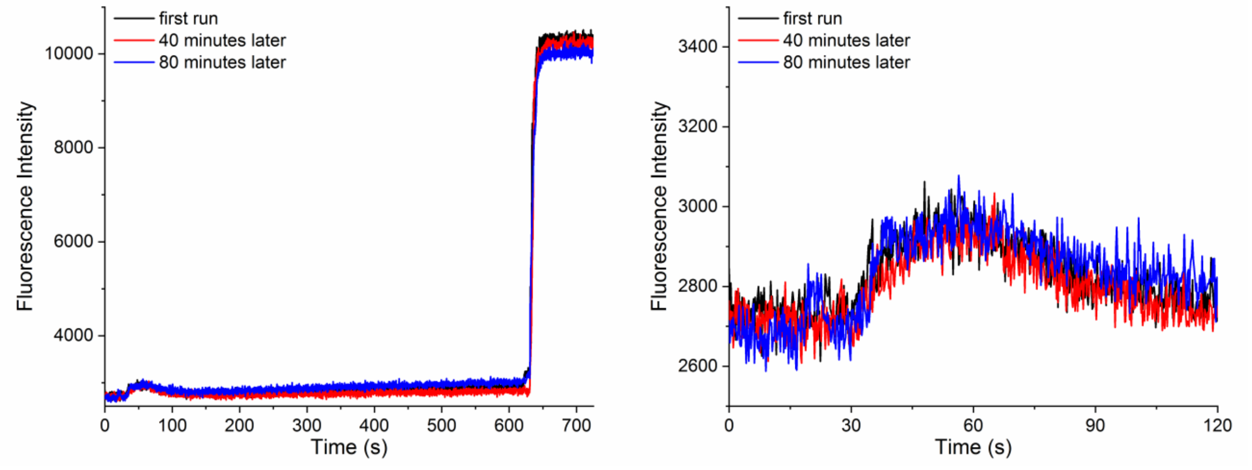

Figure S5. Three blank transport runs as monitored by the Eu.L1 assay in 225 mM NaCl with 5 mM HEPES at pH 7, upon addition of 10 mM $NaHCO_3$. The LUVs were lysed 10 minutes after the addition of $NaHCO_3$.

Compatibility of the assay with different lipids

To demonstrate that the EuL1 assay is also compatible with other lipids than POPC and cholesterol, we prepared DPPC liposomes with **1** incorporated (1:25k ratio) and tested the transport at 25°C and 45°C, which is respectively below and above the phase transition temperature of DPPC (41°C). At 25°C the DPPC lipids are in the gel phase and no transport by ionophores is expected, while the fluid phase is retrieved above 41°C, leading to a restoration of the anion transport by **1**. These results are in agreement with those obtained for Cl^-/HCO_3^- transport in the lucigenin assay.[1]

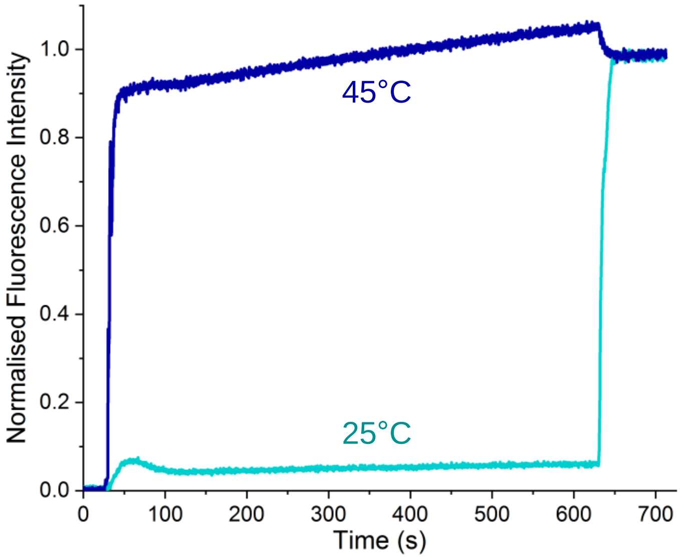

Figure S6. Transport as monitored by the Eu.L1 assay with **1** preincorporated in DPPC vesicles at 1:25k transporter to lipid ratio, below (cyan) and above (dark blue) the phase transition temperature of DPPC. The liposomes were suspended in 225 mM NaCl with 5 mM HEPES at pH 7 and 10 mM $NaHCO_3$ was added after 30 seconds and Triton X-100 was added 10 minutes after the addition of $NaHCO_3$.

2.5 Transport by monensin and other cation transporters

Transport by different cationophores and gramicidin in K_2SO_4

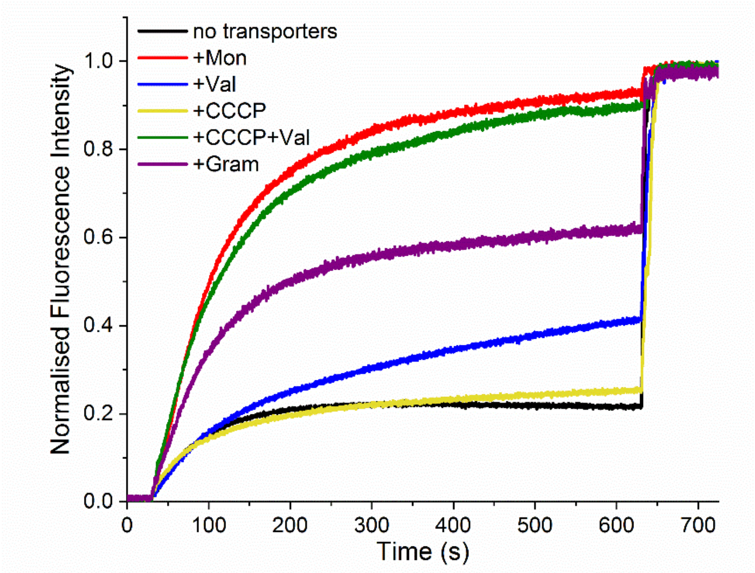

Figure S7. Transport as monitored by the Eu.L1 assay in 112 mM K_2SO_4 with 5 mM HEPES at pH 7, upon addition of 10 mM $KHCO_3$ after 30 seconds (averages of two runs per experiment). Different cation transporters were added to the LUVs 5 minutes before addition of the $KHCO_3$ at a transporter to lipid ratio of 1:1000. The LUVs were lysed 10 minutes after the addition of $KHCO_3$.

Transport by monensin in different salt solutions

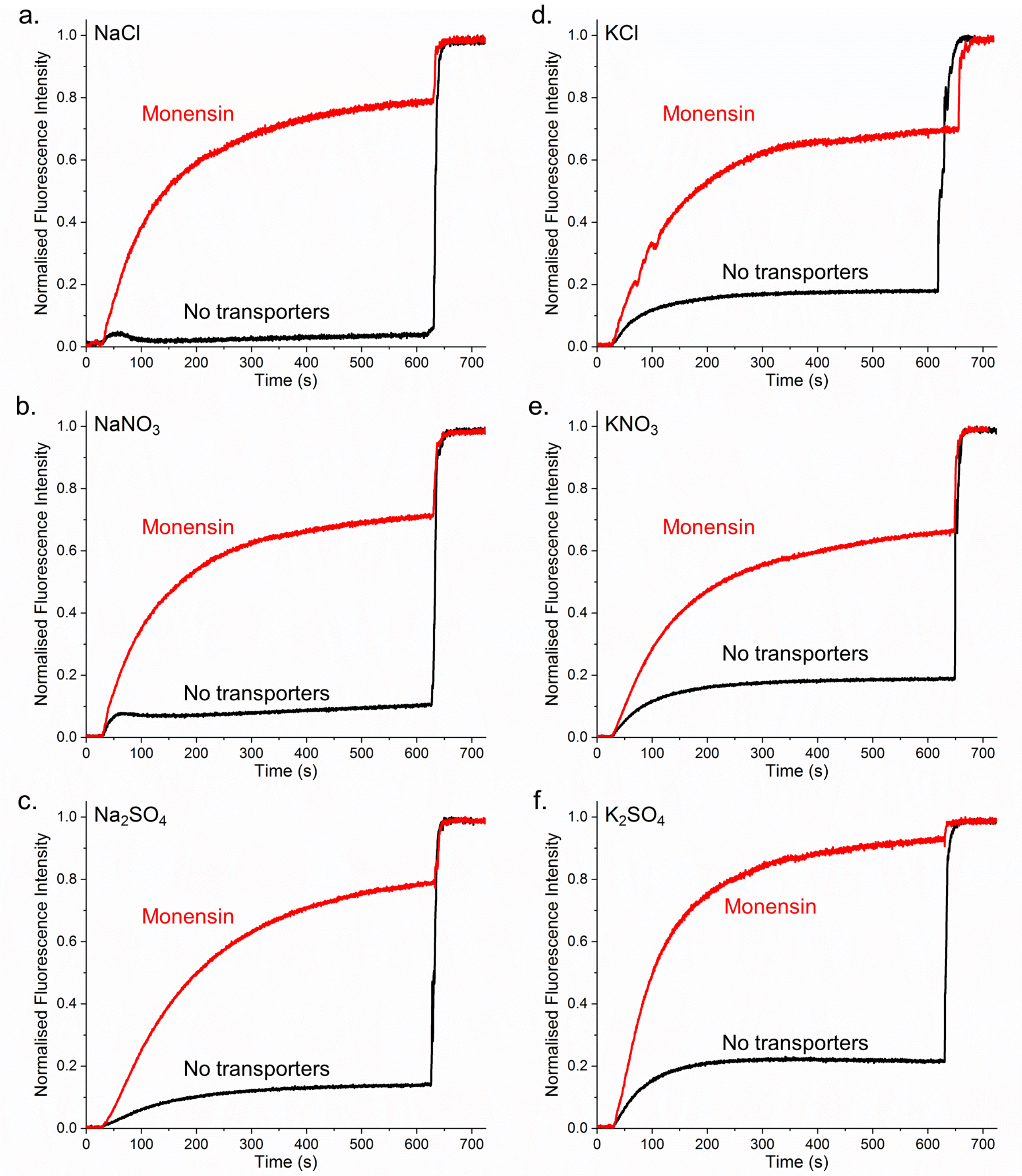

Figure S8. Transport as monitored by the Eu.L1 assay in different salt solutions (225 mM MCl, 225 mM MNO_3, or 112 mM M_2SO_4) with 5 mM HEPES at pH 7, upon addition of 10 mM $MHCO_3$ after 30 seconds. Monensin was added to the LUVs 5 minutes before addition of the $MHCO_3$ at a transporter to lipid ratio of 1:1000. The LUVs were lysed 10 minutes after the addition of $MHCO_3$.

Transport with different concentrations of monensin

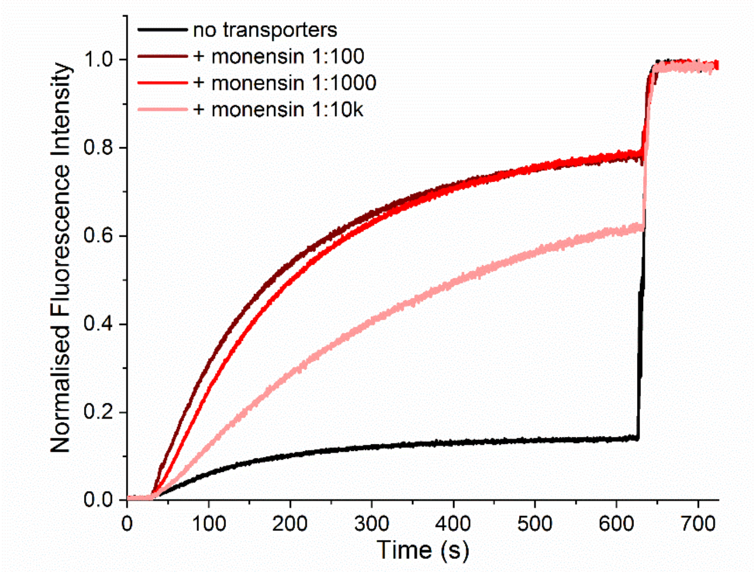

Figure S9. Transport as monitored by the Eu.L1 assay in 112 mM Na_2SO_4 with 5 mM HEPES at pH 7, upon addition of 10 mM $NaHCO_3$ after 30 seconds (averages of two runs per experiment). Different concentrations of monensin were added to the LUVs 5 minutes before addition of the $NaHCO_3$, using solutions of 2.4 mM, 0.24 mM, and 0.024 mM in methanol respectively to obtain 1:100, 1:1000, and 1:10k ratios of monensin:lipid ratios. The LUVs were lysed 10 minutes after the addition of $NaHCO_3$.

While the rate of transport by monensin at 1:10k is clearly inferior to the rates at higher concentration, the curves for 1:1000 and 1:100 can hardly be distinguished, indicating that at a monensin concentration of 1:1000 the Na^+/H^+ antiport by monensin is not rate-limiting for the overall transport process.

Transport at different concentrations of buffer and at different pH

Considering that the role of monensin in the proposed transport process is to prevent the formation of a pH gradient upon CO_2 diffusion, we may expect that in the absence of any transporters the concentration of the buffer and the pH at which the experiments are performed determine how much CO_2 can diffuse into the LUVs before the pH gradient stops this process. For this reason, we varied the starting pH and HEPES concentration.

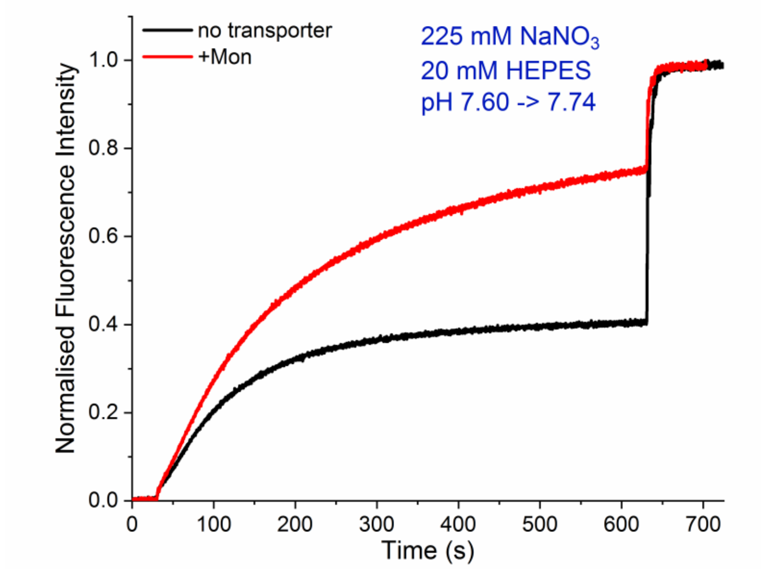

Figure S10. Transport as monitored by the Eu.L1 assay in 225 mM $NaNO_3$ with 20 mM HEPES at pH 7.6, upon addition of 10 mM $NaHCO_3$ (increasing the pH to 7.74) after 30 seconds. Monensin was added to the LUVs 5 minutes before addition of the $NaHCO_3$ at a transporter to lipid ratio of 1:1000. The LUVs were lysed 10 minutes after the addition of $NaHCO_3$. The data displayed in this Figure are single runs instead of averages.

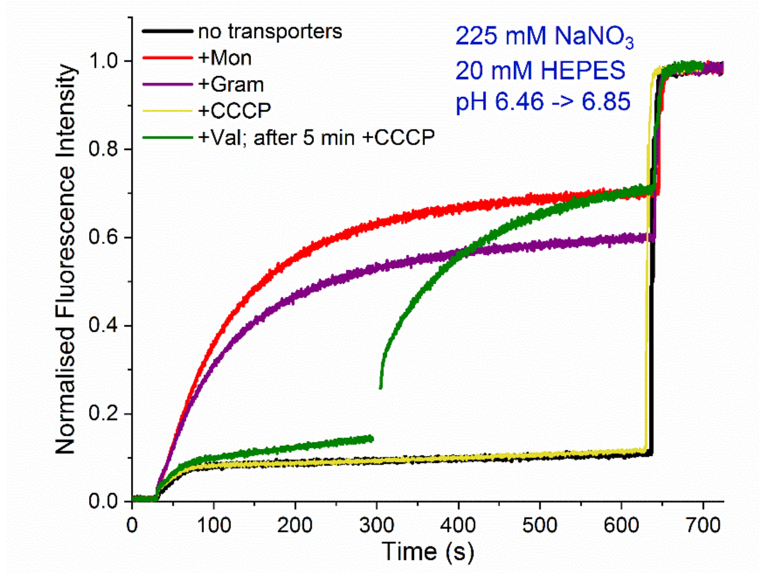

Figure S11. Transport as monitored by the Eu.L1 assay in 225 mM $NaNO_3$ with 20 mM HEPES at pH 6.5, upon addition of 10 mM $NaHCO_3$ (increasing the pH to 6.85) after 30 seconds. Different cation transporters were added to the LUVs 5 minutes before addition of the $NaHCO_3$ at a transporter to lipid ratio of 1:1000. In the experiment with valinomycin (green), 10 mM KCl was added before the start of the measurement. The LUVs were lysed 10 minutes after the addition of $NaHCO_3$. The data displayed in this Figure are single runs instead of averages.

When controls for transport experiments were performed by adding 10 mM $NaHCO_3$ to LUVs in 20 mM HEPES at a starting pH of 7.6, the external pH increased to 7.7 and a much larger apparent influx of HCO_3^- was observed (Figure S10) than when working with 5 mM HEPES at pH 7.0 (where addition of 10 mM $NaHCO_3$ increases the pH to 7.4). HEPES has a pK_a of 7.5 and the optimal buffer range is reported to be 6.8-8.2,[6] thus the higher concentration of HEPES can compensate the effect of the acidification by CO_2 influx. When 20 mM HEPES was used but starting at pH 6.5, the buffer could not compensate for the acidification and hardly any CO_2 diffusion was observed (Figure S11).

2.6 Bicarbonate transport by anionophores in NaCl solutions

Bicarbonate transport by anionophores 3 and 4 in NaCl, in absence and presence of monensin

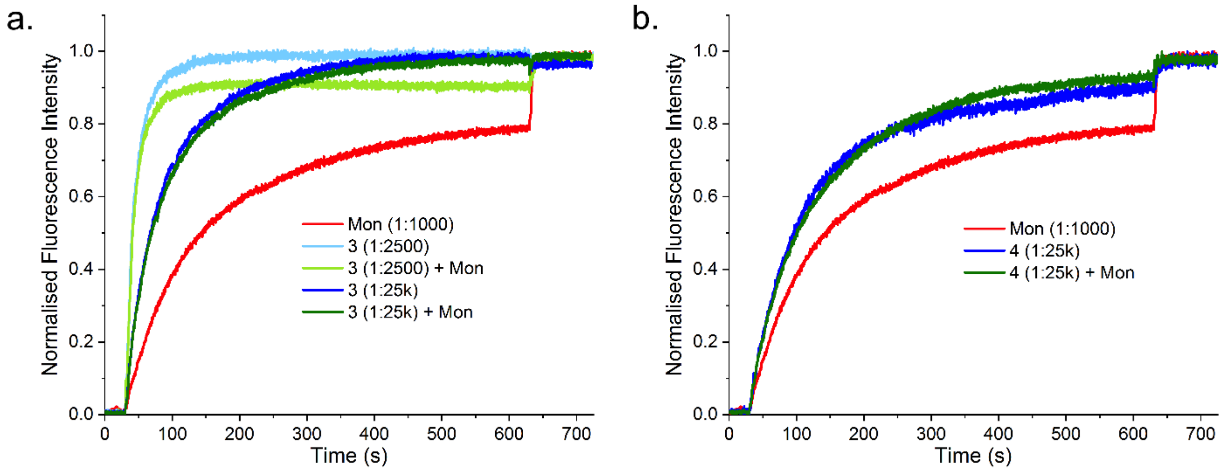

Figure S12. Transport by thiourea **3** (a) and prodigiosin (**4**,[ii] b) as monitored by the Eu.L1 assay in 225 mM NaCl with 5 mM HEPES at pH 7, upon addition of 10 mM $NaHCO_3$ after 30 seconds. Monensin was added to the LUVs at 1:1000 transporter to lipid ratio, 5 minutes before addition of the $NaHCO_3$. The LUVs were lysed after 10 minutes.

Bicarbonate transport by anionophores 1 and 2, with different concentrations of monensin

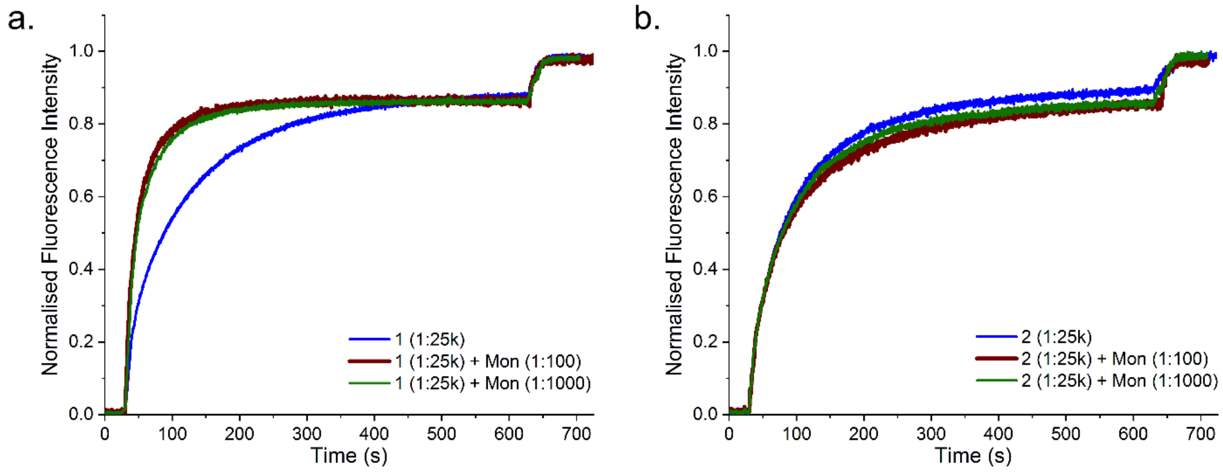

Figure S13. Transport as monitored by the Eu.L1 assay in 225 mM NaCl with 5 mM HEPES at pH 7, upon addition of 10 mM $NaHCO_3$ after 30 seconds. The LUVs were lysed 10 minutes after the addition of $NaHCO_3$. Anionophores **1** (a.) or **2** (b.) were preincorporated at 1:25k anionophore:lipid ratio. Different concentrations of monensin were added to the LUVs 5 minutes before addition of the $NaHCO_3$, using solutions of 2.4 mM and 0.24 mM in methanol respectively to obtain 1:100 and 1:1000 ratios of monensin:lipid ratios.

[ii] Data for prodigiosin **4** at 1:2500 are not reported, due to the significant fluorescence from this ionophore observed when added at such a high concentration.

HCO_3^-/Cl^- exchange by bambusuril 1, in absence and presence of monensin

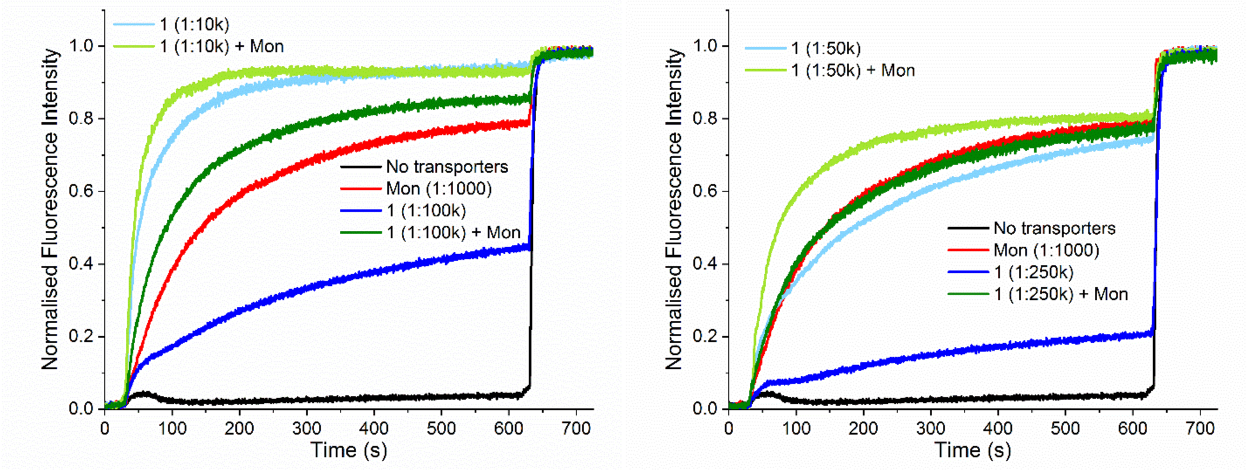

Figure S14. Transport by **1** (at different concentrations) as monitored by the Eu.L1 assay in 225 mM NaCl with 5 mM HEPES at pH 7, upon addition of 10 mM $NaHCO_3$ after 30 seconds. Monensin was added to the LUVs at 1:1000 transporter to lipid ratio, 5 minutes before addition of the $NaHCO_3$. The LUVs were lysed after 10 minutes.

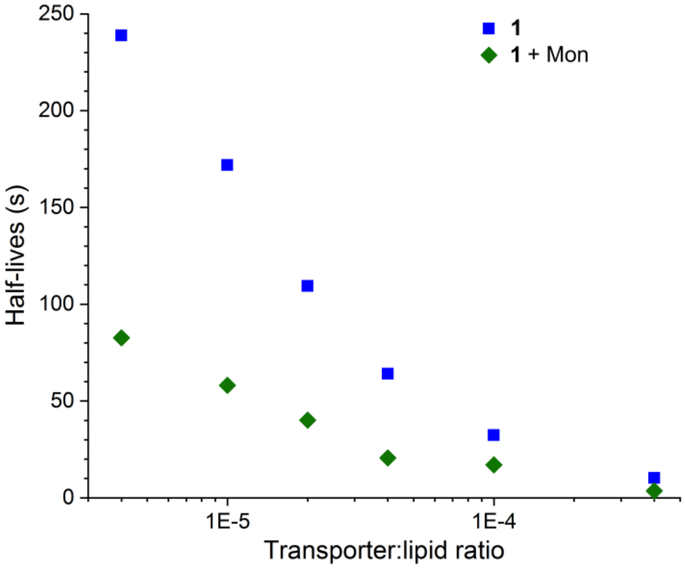

Figure S15. Plot of the half-lives obtained from the fit of the transport curves by bambusuril **1** (blue squares) versus the concentration of **1** in the membrane. A strong decrease of the half-life of HCO_3^-/Cl^- exchange is observed upon the addition of monensin to the LUVs with **1** preincorporated for all the different concentrations tested (green diamonds).

2.7 Bicarbonate transport by anionophores in $NaNO_3$ solutions

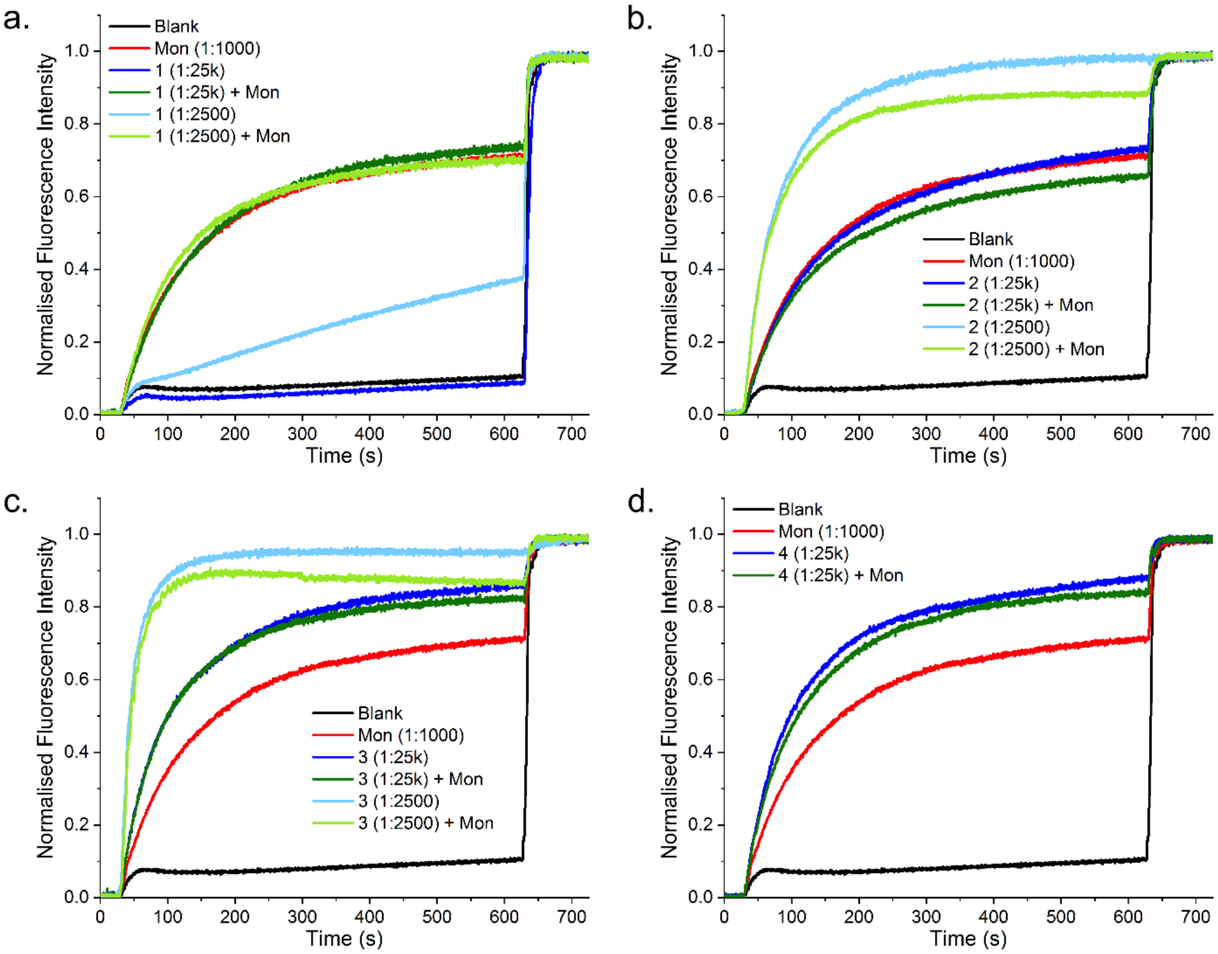

Figure S16. Transport by anionophores **1** (a.), **2** (b.), **3** (c.), and prodigiosin (**4**, d.) as monitored by the Eu.L1 assay in 225 mM $NaNO_3$ with 5 mM HEPES at pH 7, upon addition of 10 mM $NaHCO_3$ after 30 seconds. The LUVs were lysed 10 minutes after the addition of $NaHCO_3$. Monensin (1:1000 transporter to lipid ratio) was added to the LUVs 5 minutes before addition of the $NaHCO_3$.

We have also observed that HCO_3^-/Cl^- exchange by bambusuril **1** is inhibited by addition of $NaNO_3$ (Figure S17), similarly as addition of $NaNO_3$ was found to inhibit Cl^-/HCO_3^- exchange by **1** in the lucigenin assay.[1]

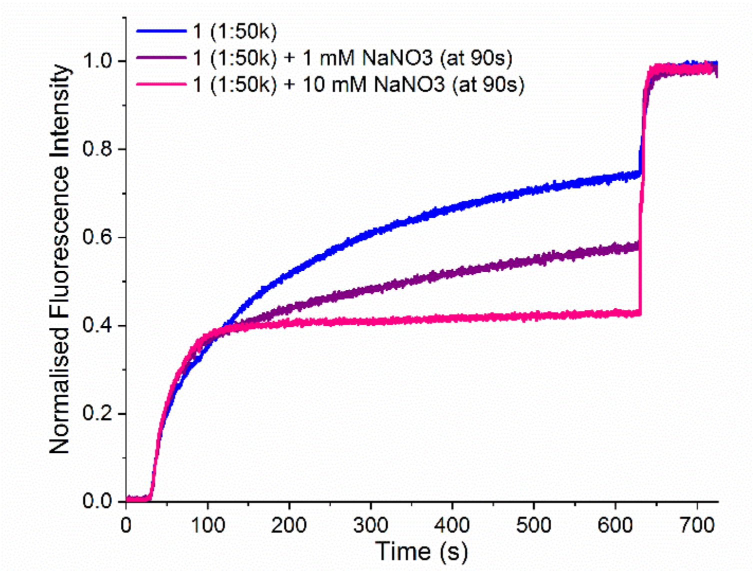

Figure S17. Transport by **1** (preincorporated at 1:50k anionophore:lipid ratio) as monitored by the Eu.L1 assay in 225 mM NaCl with 5 mM HEPES at pH 7, upon addition of 10 mM $NaHCO_3$ after 30 seconds (blue). Addition of 10 mM $NaNO_3$ after 90 s brought the HCO_3^-/Cl^- exchange to a complete halt (red), while addition of 1 mM $NaNO_3$ reduced the rate of transport. The LUVs were lysed 10 minutes after the addition of $MHCO_3$.

2.8 Bicarbonate uniport by anionophores in KGluconate solutions

The EuL1 assay was adapted to study HCO_3^- uniport by replacing the NaCl or $NaNO_3$ by 100 mM gluconic acid potassium salt (KGluc) and by addition of the cationophore valinomycin. A bicarbonate gradient was created by addition of 10 mM $KHCO_3$. Gluconate anions are too hydrophilic to participate in an antiport mechanism and instead transport of K^+ by valinomycin can balance the uniport of HCO_3^- by anionophores.[7]

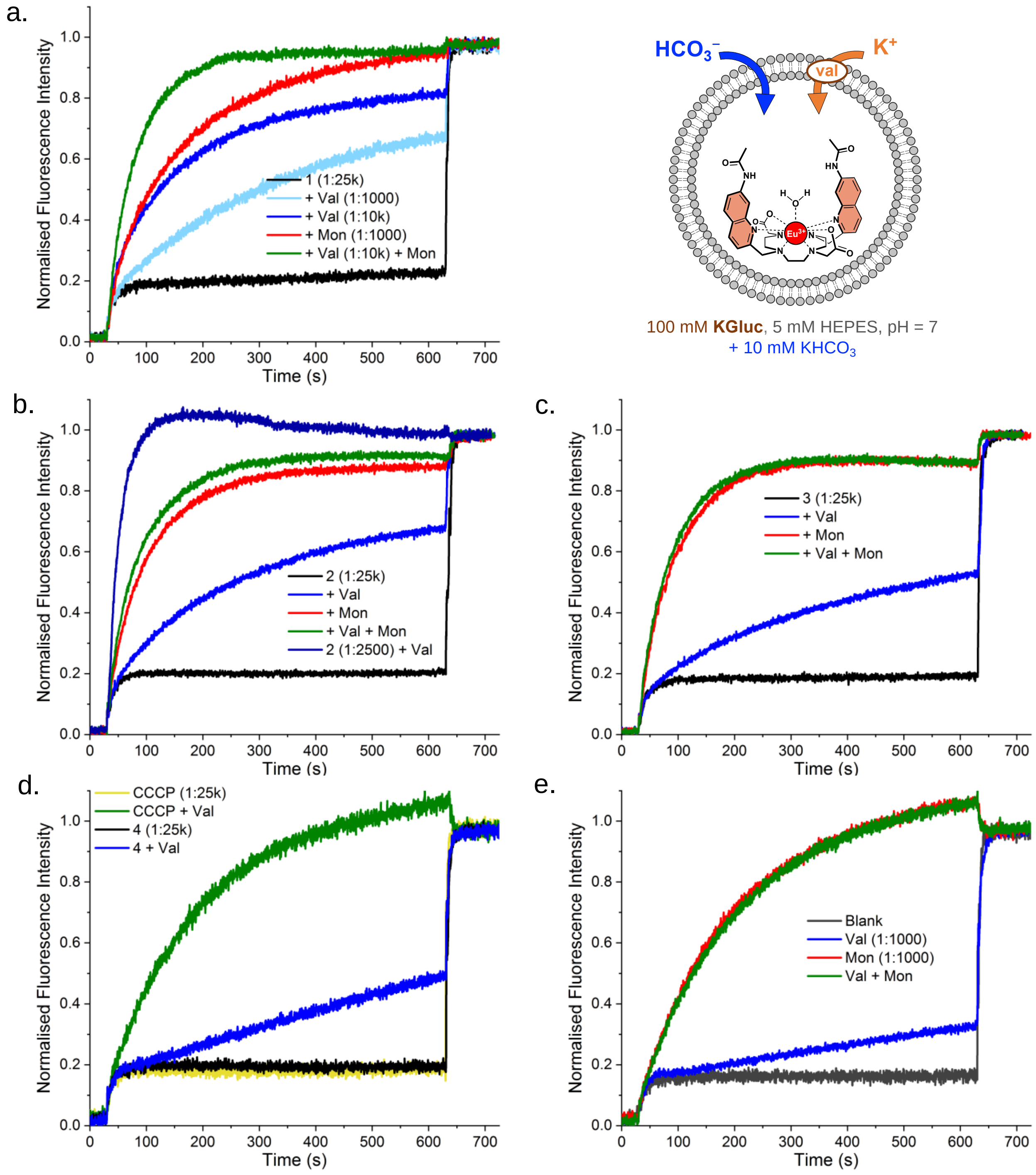

Figure S18. Transport by anionophores **1** (a.), **2** (b.), **3** (c.), (**4**, d.), and by cationophores only (e.) as monitored by the Eu.L1 assay in 100 mM KGluc with 5 mM HEPES at pH 7, upon addition of 10 mM $KHCO_3$ after 30 seconds. The LUVs were lysed 10 minutes after the addition of $KHCO_3$. Cationophores valinomycin and/or monensin were added to the LUVs 5 minutes before addition of the $KHCO_3$ at 1:1000 transporter to lipid ratio, unless indicated otherwise.[iii]

While the main results obtained from this HCO_3^- uniport assay are discussed in the main text, here we will comment on the mechanisms and effects of the different cationophores in more detail. No response is observed in the presence of anionophores **1-4** or protonophore CCCP alone, as no symport or antiport mechanisms are possible. When monensin is added (Figure S18e, red curve) or the combination of valinomycin and CCCP (Figure S18d, green curve), then an increase in the concentration of HCO_3^- is observed, which can be attributed to H^+/K^+ exchange in combination with CO_2 diffusion (mechanism B).

When combined with valinomycin, anionophores can give rise to a response in the EuL1 assay by two potential mechanism: they can either perform true HCO_3^- uniport (as indicated in the cartoon in Figure S18), or they could act a H^+ (or OH^-) transporter in a way identical to CCCP. This latter option would lead to a transport process of which the rate is limited by CO_2 diffusion, as for transport by monensin and CCCP+valinomycin. Transport exceeding this rate can thus be attributed to HCO_3^- uniport, as is the case for **1** (which is, moreover, incapable of transporting H^+ or OH^-) and for **2** at higher concentrations (1:2500), see also Table S2 on pS20.

Further insight can be obtained by combining the anionophores with monensin. While combining **1** and monensin gives only slightly faster transport than the apparent HCO_3^- transport by monensin alone, combining **2** or **3** with monensin leads to significantly faster transport. This can be explained by a mechanism in which **2** and **3** do transport the HCO_3^- anion into the liposomes, but return deprotonated (or with a OH^- anion bound), leading to HCO_3^-/H^+ symport, which would give acidification of the interior of the liposomes if the pH gradient was not dissipated by monensin. Especially thiourea **3** appears to prefer this mechanism over HCO_3^- uniport in presence of valinomycin. This is analogous to the work by Gale and co-workers on Cl^- transport in which they showed that certain anionophores preferred to couple to monensin (acting as Cl^-/H^+ symporters), while other anionophores could couple to valinomycin and perform Cl^- uniport.[8]

[iii] Valinomycin was used at a 1:1000 transporter to lipid ratio in most experiments, as higher valinomycin concentrations show a response even in absence of anionophores. While a lower valinomycin concentration (1:10k) gives lower rates of global HCO_3^- transport by compounds **2** and **3**, the opposite was found for bambusuril **1**, which gave better rates when using 1:10k than 1:1000 valinomycin. This is in line with results obtained for **1** in various conditions and assays, showing a detrimental effect of the addition of increasing amounts of valinomycin on the rates of transport by **1**. For this reason, we report the HCO_3^- uniport data for **1** with a valinomycin concentration of 1:10k.

2.9 Quantification of rates of transport

Table S1 Performance of anionophores **1**-**3** and prodigiosin **4** in the EuL1 assay in NaCl and $NaNO_3$.

Salt	Anionophore	Concentration	Transport (without monensin)		Transport (with monensin)[c]	
		(anionophore:lipid)	Half-life (s)[a]	Initial rate (s^{-1})[b]	Half-life (s)[a]	Initial rate (s^{-1})[b]
NaCl	None		*	*	82	0.008
	**1**	1:2500	10	0.124	4	0.178
		1:10k	32	0.041	17	0.059
		1:25k	64	0.026	21	0.051
		1:50k	110	0.015	40	0.025
		1:100k	*	*	58	0.015
		1:250k	*	*	83	0.009
	**2**	1:2500	12	0.093	11	0.091
		1:25k	51	0.019	50	0.017
	**3**	1:2500	12	0.077	11	0.071
		1:25k	46	0.021	47	0.020
	**4**	1:25k	59	0.012	74	0.012
$NaNO_3$	None		*	*	81	0.008
	**1**	1:2500	*	*	67	0.009
		1:25k	*	*	85	0.007
	**2**	1:2500	45	0.021	40	0.022
		1:25k	89	0.007	85	0.007
	**3**	1:2500	16	0.060	14	0.052
		1:25k	65	0.014	59	0.013
	**4**	1:25k	61	0.012	68	0.011

[a] Calculated from a single exponential fit of the transport curve, see ESI for details.
[b] Calculated from a double exponential fit of the transport curve, see ESI for details.
[c] Transport in presence of monensin at a 1:1000 monensin to lipid ratio.
* Transport was absent or too slow to quantify.

Based on the discussion of the additivity of mechanisms A and B, we could have expected that initial rates of transport by **1** (at different concentrations) and by monensin alone (1:1000 ratio) could be added to predict the initial rates of transport by the combination of **1** and monensin. However, in Table S1 we see that the values found are higher than those predicted by this hypothesis, especially at higher concentrations of **1** (≥ 1:25k ratio).

When comparing initial rates of transport as measured in the EuL1 assay quantitively, we should consider the effect of the different pH profiles during the transport measurements (Fig. 6d-f), as the $[Eu.L^1]^+$ probe is not only sensitive to concentrations of HCO_3^-, but also to pH.[4] While the effects of pH on the emission intensity were reported to be small at pH ≤ 8, the pH has a more

noticeable effect on the increase of luminescence observed upon addition of HCO_3^-. The titrations in Figure S19 and Figure S20 (see next Section) show that this increase of emission intensity is larger at pH 7.4 than at pH 7.0, leading to different apparent affinities of EuL1 for HCO_3^-. In the presence of monensin, the pH rapidly equilibrates to ~7.4 (Fig. 6e, green curve), thus resulting in a higher sensitivity of the probe to HCO_3^-, which could result in a higher apparent initial rate compared to transport measured under conditions without pH equilibration (*i.e.*, with only **1** present in the LUVs).

Furthermore, the normalisation of the data could result in an error on the values of the initial rates, but not the half-lives. Therefore, half-lives are more reliable to compare transport data of **1** in presence and absence of monensin, as these values indicate how fast equilibrium is reached, independent of absolute emission values. The comparison of half-lives of transport by **1** with and without monensin clearly shows that equilibrium is reached much faster in the presence of monensin (Table S1 and Figure S15), confirming the additivity of mechanisms A and B.

For the HCO_3^- uniport studies in KGluc solution, we determined the half-lives in presence of valinomycin (typical HCO_3^- uniport experiment), but also in presence of monensin and both monensin and valinomycin. See page S19 for a discussion of these data.

Table S2 Half-lives for HCO_3^- uniport by anionophores **1**-**4** in the EuL1 assay in KGluc.

**Anionophore**	**Concentration (anionophore: lipid)**	**Half-life (s)** ***with valinomycin***	**Half-life (s)** ***with monensin***	**Half-life (s)** ***with valinomycin and monensin***
None			117	124
**1**	1:25k	83	89	38
**2**	1:25k	138	52	45
**2**	1:2500	14	14	10
**3**	1:25k	178	47	42
**4**	1:25k	>600	nd	nd
**CCCP**	1:25k	111	nd	nd

nd = Not determined

2.10 Titrations of Eu.L1 with $NaHCO_3$

Titrations of $[Eu.L^1]^+$ probe with $NaHCO_3$ were performed to study the combined effect of the pH and the HCO_3^- concentration on the emission intensity. Emission spectra were recorded on a Fluoromax-4 spectrometer at 25 °C from 550-725 nm (1 nm slits, 1 nm interval, 0.3 s integration time) with excitation at 330 nm (1 nm slits) and with a 420 nm cut-off filter between the sample and the detector.

Titration under standard transport conditions (Sample 1)

To 1.5 mL of a solution of $[Eu.L^1]^+$ probe (50 µM) in the adequate buffer (225 mM NaCl, 5 mM HEPES, **pH 7.0**) aliquots of 0.8 M $NaHCO_3$ (3.8 – 19 µL; in 225 mM NaCl) were added, and after each addition the sample was homogenized and the emission spectrum recorded. The pH of the sample increased progressively from 7.0 to 7.9 (after addition of 30 mM $NaHCO_3$).

Titration at pH higher than the standard (Sample 2)

To 1.5 mL of a solution of $[Eu.L^1]^+$ probe (50 µM) in the adequate buffer (225 mM NaCl, 5 mM HEPES, **pH 7.4**) aliquots of 0.8 M $NaHCO_3$ (3.8 – 19 µL; in 225 mM NaCl) were added, and after each addition the sample was homogenized and the emission spectrum recorded. The pH of the sample increased progressively from 7.4 to 8.0 (after addition of 30 mM $NaHCO_3$).

Titration at constant pH (Sample 3)

A solution of $[Eu.L^1]^+$ probe (50 µM) in the adequate buffer (225 mM NaCl, **100 mM HEPES**, pH 7.4) was degassed by bubbling argon for 1 h, to remove any atmospheric CO_2 that could be dissolved due to the high concentration of buffer. To 1.5 mL of this solution aliquots of 0.8 M $NaHCO_3$ (3.8 – 20 µL; in 225 mM NaCl) were added, and after each addition the sample was homogenized and the emission spectrum recorded. The pH of the sample was maintained at 7.4-7.5 (after addition of 30 mM and 40 mM $NaHCO_3$, 10 µL of 0.5 M HCl were added to maintain the pH into the desired range).

Data treatment

The values of fluorescence intensity measured at 615 nm during the titrations were corrected by the factor of dilution resulting from the additions of $NaHCO_3$ and HCl solutions (assuming that under the conditions of the experiment the fluorescence intensity has a linear dependence on the concentration of $[Eu.L^1]^+$). The results are presented in Figure S19a and show a significant dependence of the increase in fluorescence intensity on the pH of the sample. This is illustrated in Figure S19b, where the emission spectra of samples 1 and 2 show only small differences in absence of $NaHCO_3$, while upon addition of 4 mM $NaHCO_3$ (data points in the grey box in Figure S19a) a significant difference in intensity is observed.
The corrected titration data have also been fitted to a 1:1 binding model[9] in Origin 9.55, showing a significant variation in apparent affinity constants of $[Eu.L^1]^+$ for HCO_3^- (Figure S20).

The differences observed here should be taken into consideration when quantitatively comparing data from transporters that give fast pH equilibration (a situation mimicked in sample 3) to those where the pH variation is only caused by the transport of HCO_3^- (as by bambusuril **1**, mimicked with sample 1).

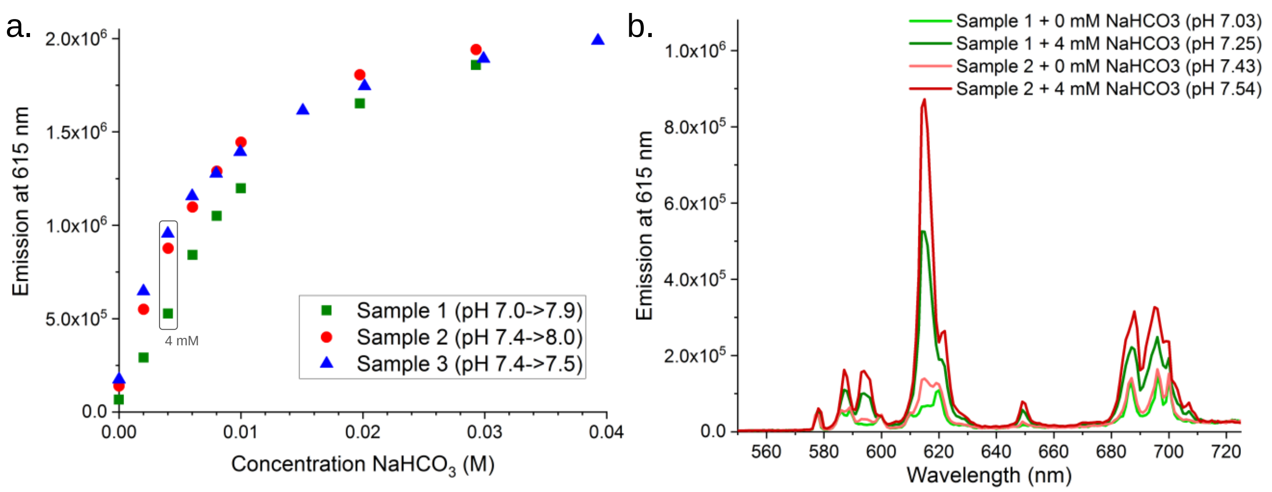

Figure S19. (a) Changes in emission intensity at 615 nm upon addition of $NaHCO_3$ to three different samples of $[Eu.L^1]^+$ (50 µM). (b) Comparison of the emission spectra of $[Eu.L^1]^+$ (50 µM) in the absence of HCO_3^- and after the addition of 4 mM $NaHCO_3^-$ for two samples at different pH, corresponding to the datapoint in the grey box in (a). Conditions: NaCl (225 mM), HEPES (5 mM for samples 1 and 2 and 100 mM for sample 3), λ_{ex} = 330 nm, 25 °C.

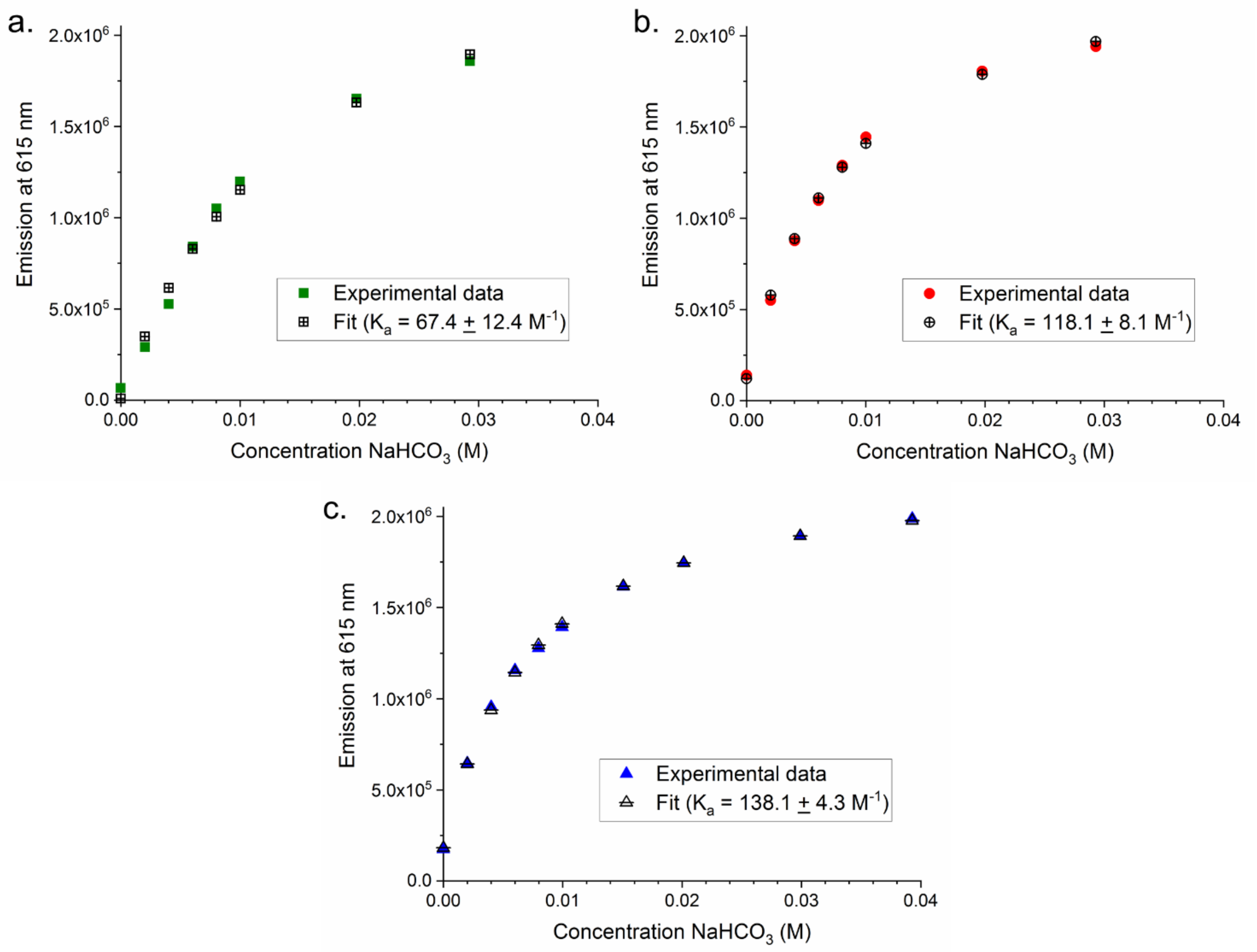

Figure S20. Fits of experimental data to a binding model that considers the formation of a 1:1 complex for samples 1 (a), 2 (b) and 3 (c).

3 Transport experiments with HPTS

3.1 Procedures for the transport experiments with HPTS

The liposomes used in these experiments were prepared as described for the Eu.L1 assay, but using a solution of 8-hydroxypyrene-1,3,6-trisulfonic acid trisodium (HPTS, 0.1 mM) in the desired buffer (225 mM NaCl, 5 mM HEPES, pH 7.0) to hydrate the lipid films.

3.00 mL of this liposome solution was placed in a quartz cuvette with a small stir bar and the temperature was allowed to stabilize at 25˚C for 3-5 min. inside the sample compartment of a Fluoromax-4 spectrometer. During transport measurements the fluorescence intensity at 511 nm (2 nm slits) was monitored over time (15 minutes, 2 s interval) for two different excitation wavelengths, 403 and 455 nm (2 nm slits).

In experiments with monensin or prodigiosin, these were added to the liposomes as 5 µL of solution in methanol 5 minutes before the addition of the bicarbonate.

For transport measurements 38 µL of $NaHCO_3$ (0.8 M, in 225 mM salt solution) was added to the liposomes 30 s after the start of the fluorescence recording, to create a HCO_3^- concentration gradient of 10 mM and a pH gradient of 7.0 (inside) to 7.5 (outside). The fluorescence intensity was measured for another 10 minutes, followed by lysing of the liposomes by addition of 50 µL of Triton X-100 (5% $^w/_w$ in water).

The transport curves were obtained from the ratio of the emission intensities from the two different excitation wavelengths (F_{455}/F_{403}) and the average of three runs was calculated. The averaged data were normalised from 0 (before the addition of $NaHCO_3$) to 1 (after lysis).

3.2 Additional results for the transport experiments with HPTS

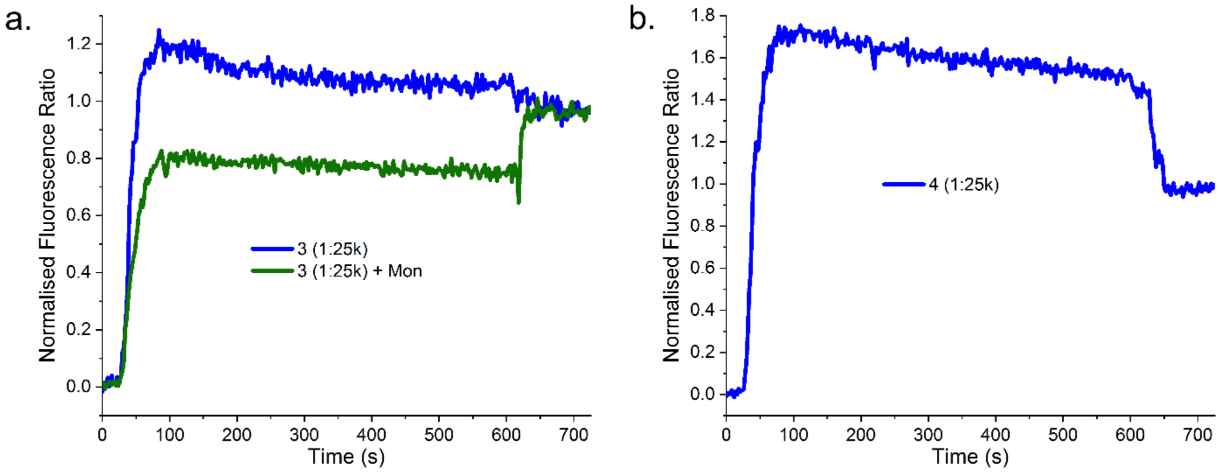

Figure S21. Transport by thiourea **3** (a) and prodigiosin (**4**, b) as monitored by HPTS in 225 mM NaCl with 5 mM HEPES at pH 7, upon addition of 10 mM $NaHCO_3$ after 30 seconds. Monensin was added to the LUVs with **3** at 1:1000 transporter to lipid ratio, 5 minutes before addition of the $NaHCO_3$. The LUVs were lysed after 10 minutes.

4 Transport experiments with ^{13}C NMR spectroscopy

Transport experiments on isotopically labelled $H^{13}CO_3^-$ were performed as described in reference 10. The following buffer solutions were prepared:

- 450 mM NaCl and 20 mM HEPES at pH 7.3 in D_2O/H_2O (1:9)
- 150 mM Na_2SO_4 and 20 mM HEPES at pH 7.3 in D_2O/H_2O (1:9).

Lipid films of EYPC were prepared from chloroform, and dried for at least 1 h under high vacuum. The EYPC lipid film (69 mg) was then hydrated with 1 mL NaCl buffer solution, containing 450 mM NaCl and 20 mM HEPES at pH 7.3 in D_2O/H_2O (9:1), resulting in a lipid concentration of 90 mM. The mixture of lipid and buffer was vortexed for 5 minutes, and the resulting vesicles were frozen and thawed 5 times and extruded 41 times through a polycarbonate membrane with 5 µm pores in a mini-extruder (Avestin LiposoFast-Basic).

^{13}C NMR spectra were recorded on a Jeol JNM-ECZ600R/S3 Spectrometer with a 5 mm Royal Probe at 25˚C, using a 30˚ pulse, an acquisition time of 0.87 s, relaxation delay of 0.2 s and 160 scans per spectrum.

For the transport experiments, 230 µL of vesicles were combined with 340 µL Na_2SO_4 buffer in an NMR tube and 38 µL of 0.8 M $NaH^{13}CO_3$ in D_2O/H_2O (1:9) was added to obtain a $NaH^{13}CO_3$ concentration of 50 mM. The ^{13}C signal of HCO_3^- was clearly observed at 160 ppm (Figure S22, red spectra). Then 8.8 µL of 35 mM $MnCl_2$ in D_2O/H_2O (1:9) was added, resulting in an overall Mn^{2+} concentration of 0.5 mM, which resulted in a broadening of the HCO_3^- signal (Figure S22, yellow spectrum). Subsequent addition of 8.65 µL of 24 mM monensin in methanol (1:1000 monensin to lipid ratio) resulted in a re-appearance of a sharp HCO_3^- signal (Figure S22a, green spectra), while this did not happen when methanol without monensin was added (Figure S22b). This small but sharp HCO_3^- signal indicates that HCO_3^- is present inside the vesicles, where the signal is not affected by the paramagnetic Mn^{2+}. Upon lysis of the vesicles by addition of 30 µL Triton X-100, this sharp interior HCO_3^- signal disappeared again (Figure S22, purple spectra).

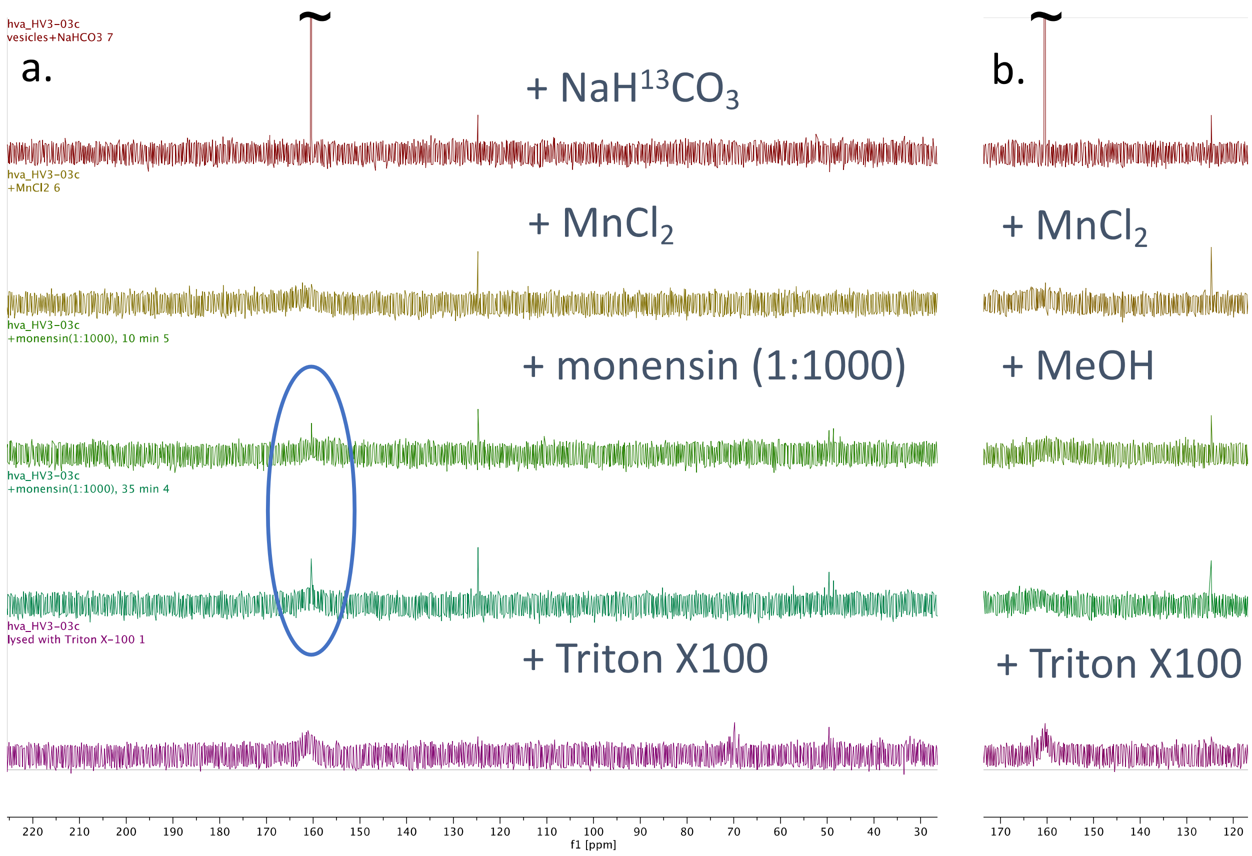

Figure S22. ^{13}C NMR spectra of vesicles with NaCl interior and $NaH^{13}CO_3$ and exterior (red), followed by addition of paramagnetic $MnCl_2$ (yellow), upon addition of monensin in methanol (a) or methanol only (b) after 10 minutes and 35 minutes (light and dark green), and upon lysis of the vesicles with Triton X-100 (purple). The appearance of a sharp HCO_3^- signal after the addition of monensin indicates that HCO_3^- has been transferred to the interior of the vesicles.

5 References

[1] H. Valkenier, O. Akrawi, P. Jurček, K. Sleziaková, T. Lízal, K. Bartik and V. Šindelář, *Chem*, 2019, **5**, 429–444.

[2] S. Hussain, P. R. Brotherhood, L. W. Judd and A. P. Davis, *J. Am. Chem. Soc.*, 2011, **133**, 1614–1617.

[3] H. Valkenier, L. W. Judd, H. Li, S. Hussain, D. N. Sheppard and A. P. Davis, *J. Am. Chem. Soc.*, 2014, **136**, 12507–12512.

[4] S. J. Butler, *Chem. Commun.*, 2015, **51**, 10879–10882.

[5] H. Ozbek, J. A. Fair and S. L. Phillips, Viscosity of Aqueous Sodium Chloride Solutions From 0 150° C, 1977 https://digital.library.unt.edu/ark:/67531/metadc1014165/, (accessed June 9, 2020).

[6] "Biological Buffers," in CRC Handbook of Chemistry and Physics, 100th Edition (Internet Version 2019), John R. Rumble, ed., CRC Press/Taylor & Francis, Boca Raton, FL.

[7] L. A. Jowett, E. N. W. Howe, X. Wu, N. Busschaert and P. A. Gale, *Chem. Eur. J.*, 2018, **24**, 10475–10487.

[8] X. Wu, L. W. Judd, E. N. W. Howe, A. M. Withecombe, V. Soto-Cerrato, H. Li, N. Busschaert, H. Valkenier, R. Pérez-Tomás, D. N. Sheppard, Y.-B. Jiang, A. P. Davis and P. A. Gale, Chem, 2016, **1**, 127–146.

[9] P. Thordarson, *Chem. Soc. Rev.*, 2011, **40**, 1305–1323.

[10] J. T. Davis, P. A. Gale, O. A. Okunola, P. Prados, J. C. Iglesias-Sánchez, T. Torroba and R. Quesada, *Nat. Chem.*, 2009, **1**, 138–144.
